# A New Sauropodomorph Dinosaur from the Early Jurassic of Patagonia and the Origin and Evolution of the Sauropod-type Sacrum

**DOI:** 10.1371/journal.pone.0014572

**Published:** 2011-01-26

**Authors:** Diego Pol, Alberto Garrido, Ignacio A. Cerda

**Affiliations:** 1 Consejo Nacional de Investigaciones Científicas y Técnicas (CONICET), Museo Paleontológico Egidio Feruglio, Trelew, Argentina; 2 Museo Provincial de Ciencias Naturales “Prof. Dr. Juan A. Olsacher”, Zapala, Argentina; 3 CONICET-INBIOMA, Museo de Geología y Paleontología, Universidad Nacjonal del Comahue, Neuquén, Argentina; Raymond M. Alf Museum of Paleontology, United States of America

## Abstract

**Background:**

The origin of sauropod dinosaurs is one of the major landmarks of dinosaur evolution but is still poorly understood. This drastic transformation involved major skeletal modifications, including a shift from the small and gracile condition of primitive sauropodomorphs to the gigantic and quadrupedal condition of sauropods. Recent findings in the Late Triassic–Early Jurassic of Gondwana provide critical evidence to understand the origin and early evolution of sauropods.

**Methodology/Principal Findings:**

A new sauropodomorph dinosaur, *Leonerasaurus taquetrensis* gen. et sp. nov., is described from the Las Leoneras Formation of Central Patagonia (Argentina). The new taxon is diagnosed by the presence of anterior unserrated teeth with a low spoon-shaped crown, amphicoelous and acamerate vertebral centra, four sacral vertebrae, and humeral deltopectoral crest low and medially deflected along its distal half. The phylogenetic analysis depicts *Leonerasaurus* as one of the closest outgroups of Sauropoda, being the sister taxon of a clade of large bodied taxa composed of *Melanorosaurus* and Sauropoda.

**Conclusions/Significance:**

The dental and postcranial anatomy of *Leonerasaurus* supports its close affinities with basal sauropods. Despite the small size and plesiomorphic skeletal anatomy of *Leonerasaurus*, the four vertebrae that compose its sacrum resemble that of the large-bodied primitive sauropods. This shows that the appearance of the sauropod-type of sacrum predated the marked increase in body size that characterizes the origins of sauropods, rejecting a causal explanation and evolutionary linkage between this sacral configuration and body size. Alternative phylogenetic placements of *Leonerasaurus* as a basal anchisaurian imply a convergent acquisition of the sauropod-type sacrum in the new small-bodied taxon, also rejecting an evolutionary dependence of sacral configuration and body size in sauropodomorphs. This and other recent discoveries are showing that the characteristic sauropod body plan evolved gradually, with a step-wise pattern of character appearance.

## Introduction

Sauropods are one of the most recognizable groups of dinosaurs, characterized by their gigantic size, quadrupedal stance, and extremely long cervical and caudal regions of the vertebral column. These are among the most noticeable features of the sauropod body plan, which was maintained relatively unchanged during their success as the dominant herbivores of the Jurassic and Cretaceous [1]–[3]. It has long been recognized that sauropods evolved from the much smaller, gracile, and bipedal primitive sauropodomorphs, a paraphyletic assemblage of taxa previously known as ‘prosauropods’ [1], [4]–[9]. However, the evolutionary origins of sauropods are still poorly understood and, until recently, a major morphological gap separated the characteristic sauropods from the assemblage of basal sauropodomorphs.

A series of recently described forms from the Late Triassic–Early Jurassic of Gondwana have been interpreted either as sauropod outgroups or basal sauropods [10]–[14]. These have partially filled this gap and contributed to understanding the evolutionary origins of sauropods.

Recent work in the Las Leoneras Formation in Central Patagonia resulted in the discovery of partially articulated remains of a new sauropodomorph dinosaur, *Leonerasaurus taquetrensis* gen. et sp. nov., that fill an important gap in the evolutionary history of Sauropodomorpha. Although numerous characters indicate *Leonerasaurus* is a small non-sauropod sauropodomorph, details of its dental and pelvic anatomy suggest this taxon is more derived than most ‘prosauropods’ and is one of the closest outgroups of Sauropoda.

In the present contribution we describe this specimen, its geological provenance, analyze its phylogenetic relationships, and discuss the implications of its anatomy for understanding the evolutionary origin of Sauropoda, with particular emphasis on the pattern of character acquisition in the evolution of the sacrum and body size in Sauropodomorpha.

## Methods

### Terminology

#### Taxonomic nomenclature and comparisons

The comparisons made with basal sauropodomorphs and sauropods in the text are based on the examination of specimens of different taxa and relevant literature detailed in Table 1. Unless noted explicitly, all references to other taxa are based on those sources of data listed in Table 1.

**Table 1:** Source of comparative data used in this study.

Taxon	Source
*Anchisaurus polyzelus*	YPM 1883
*Antetonitrus ingenipes*	BPI/1/4952
*Coloradisaurus brevis*	PVL 5904
*Lessemsaurus sauropoides*	PVL 4822
*Lufengosaurus huenei*	IVPP V15
**Massospondylus carinatus**	BPI/1/4934
**Melanorosaurus readi**	NM QR3314
**Plateosaurus engelhardti**	SMNS 13200
*Riojasaurus incertus*	PVL 3808
*Saturnalia tupiniquim*	MCP 3844-PV
**Tazoudasaurus naimi**	Allain and Aquesbi [51]
*Yunnanosaurus huangi*	NGMJ 004546 (after Barrett et al. [49])

All comparative references to the following taxa have been observed in the listed specimens or taken from the respective bibliographic reference. Comparisons based on other specimens or taken from additional references are explicitly indicated in the text.

BPI, Bernard Price Institute, Johannesburg, South Africa; IVPP, Institute of Vertebrate Paleontology and Paleoanthropology, Beijing, People's Republic of China; MB, Institut für Palaontologie, Museum fur Naturkunde, Humbolt-Universität, Berlin, Germany; MCP, Museu Pontifícia Universidade Católica, Porto Alegre, Brazil; NGMJ, Nanjing Geological Museum, Nanjing, People's Republic of China; NM QR, National Museum, Bloemfontein, South Africa; PVL, Instituto Miguel Lillo, Tucumán, Argentina; SAM, Iziko - South African Museum, Cape Town, South Africa; SMNS, Staatliches Museum für Naturkunde Stuttgart, Stuttgart, Germany; YPM, Yale Peabody Museum, New Haven, Connecticut, USA.

Several clades names are mentioned throughout the text and their usage follows the recent literature: Sauropodomorpha [15], Anchisauria [16], Massopoda [17], Sauropoda [12], and Eusauropoda [18]. The definition of Sauropoda is the only one that has varied in recent years and for which there is no general consensus. Two recent definitions given by Sereno [19] and Yates [12] are the ones that most closely match the traditional taxonomic content of Sauropoda in phylogenetic hypotheses depicting ‘prosauropods’ as paraphyletic. We follow Yates [12], given that in his definition *Melanorosaurus* is depicted as an external specifier of Sauropoda, which is consistent with the traditional exclusion of this taxon from Sauropoda.

### Nomenclatural Acts

The electronic version of this document does not represent a published work according to the International Code of Zoological Nomenclature (ICZN), and hence the nomenclatural acts contained in the electronic version are not available under that Code from the electronic edition. Therefore, a separate edition of this document was produced by a method that assures numerous identical and durable copies, and those copies were simultaneously obtainable (from the publication date noted on the first page of this article) for the purpose of providing a public and permanent scientific record, in accordance with Article 8.1 of the Code. The separate print-only edition is available on request from PLoS by sending a request to PLoS ONE, 185 Berry Street, Suite 3100, San Francisco, CA 94107, USA along with a check for $10 (to cover printing and postage) payable to “Public Library of Science”.

In addition, this published work and the nomenclatural acts it contains have been registered in ZooBank, the proposed online registration system for the ICZN. The ZooBank LSIDs (Life Science Identifiers) can be resolved and the associated information viewed through any standard web browser by appending the LSID to the prefix “http://zoobank.org/”. The LSID for this publication is: urn:lsid:zoobank.org:pub:05E09F91-864D-4C77-8164-97FEE113375A

### Phylogenetic Methods

The phylogenetic analysis aims to test the phylogenetic affinities of the new sauropodomorph described here. The dataset includes sauropodomorph outgroups (including theropods, ornithischians, and dinosauriforms), a large sample of basal sauropodormorphs, and basal sauropods. Additionally, some derived members of Eusauropoda were also included to represent the ingroup relationships of this clade. The broad scope of the taxon-sampling regime used here conforms to the general lack of consensus on the phylogenetic relationships of basal sauropodomorphs in recent phylogenetic analyses (see below and Appendix S1 for further data on the phylogenetic analysis).

The phylogenetic analysis was conducted using equally weighted parsimony in TNT v. 1.0 [20]–[21]. A heuristic tree search strategy was conducted performing 1000 replicates of Wagner trees (using random addition sequences) followed by TBR branch swapping (holding 10 trees per replicate). The best trees obtained at the end of the replicates were subjected to a final round of TBR branch swapping. Zero-length branches were collapsed if they lack support under any of the most parsimonious reconstructions (i.e., rule 1 of Coddington and Scharff [22]). Branch support of clades was evaluated by examining the most parsimonious trees in which the monophyly of a given group is rejected [23] and using both standard absolute frequencies and GC frequencies [24] in one thousand replicates of bootstrap and jackknife analysis (see Appendix S1 for further information). Some alternative phylogenetic hypotheses (placing the new taxon in alternative positions among Sauropodomorpha) have been tested through the use of monophyly constraints in TNT and the Templeton test [25].

Unstable taxa and the causes of instability were identified using the IterPCR procedure [26] over the entire set of most parsimonious trees (MPTs). The unstable taxa *Camelotia, Blikanasaurus*, *Jingshanosaurus*, and *Ferganasaurus* were pruned from the MPTs (a posteriori of the heuristic tree searches) to construct a reduced strict consensus, provide diagnosis of some relevant clades collapsed in the complete strict consensus, and evaluate nodal support (given that the alternative positions of the unstable taxa creates a minimal bound for the support of several tree nodes; see [27]). The exclusion of these taxa therefore allows a comparison of differences in branch support irrespective of their alternative positions within Sauropodomorpha.

## Results

### Geological Setting

**Figure 1:**
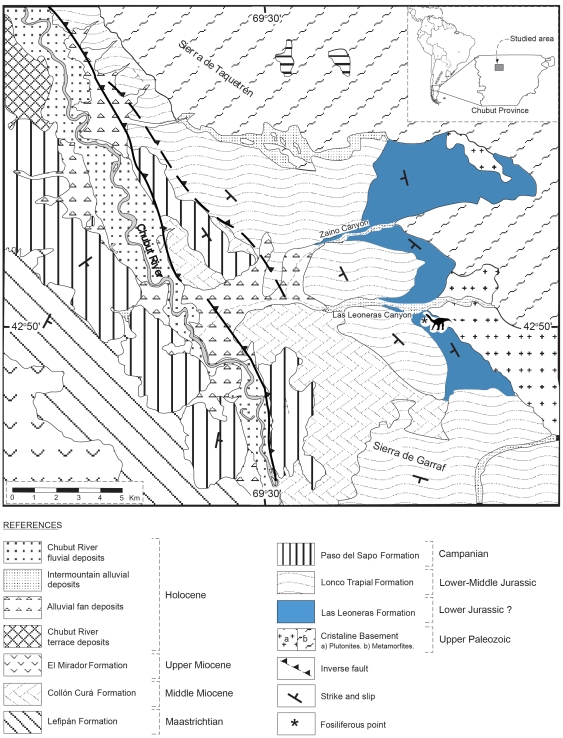
Geological map of the locality where *Leonerasaurus taquetrensis* was found (indicated by asterisk and silhouette).

The dinosaur remains were recovered from the uppermost part of the Las Leoneras Formation (Figure 1), a sequence of continental deposits of presumed Lower Jurassic age briefly described by Nakayama [28]. This unit was deposited onto the paleorelief of granitic rocks of the Mamil Choique Formation (Lower Ordovician), and is unconformably covered by andesite and volcaniclastic deposits of the Lonco Trapial Formation (Middle Jurassic; [28]–[35]).

**Figure 2:**
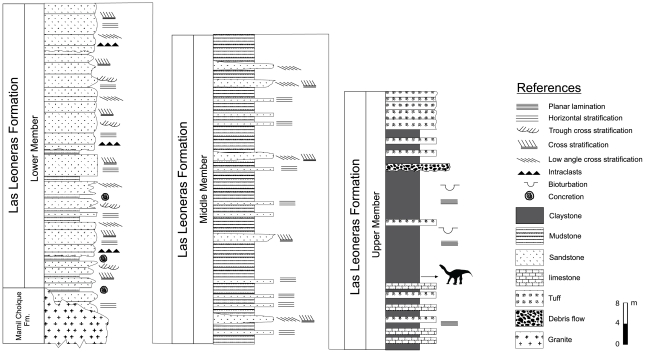
Geological section of Las Leoneras Formation. A detailed section of the three members recognized here for the Las Leoneras Formation is given, starting from the base (left of the figure) to the top of the unit (right of the figure).

Three members are here recognized for the Las Leoneras Formation (Figure 2) in the stratigraphic section taken at the type locality. The total measured thickness for this unit is 179.5 m. The Lower Member is represented by 59 m of white, medium and coarse-grained, poorly to moderately sorted sandstones; with thin and scattered intercalations of purple, massive, sandy mudstones. The sandstone levels are dominated by poorly rounded clasts of quartz, plagioclase, and biotite, showing an identical composition to the underlying rocks of the Mamil Choique Formation. The Lower Member is characterized by amalgamated channelized bodies, with a predominance of planar cross-stratification and horizontal stratification. Lag deposits, intraclasts, and fine conglomeratic lenses are common in the base of the paleochannels. The sedimentological characteristics of these beds suggest that these fluvial deposits were generated by gravel-sandy braided systems [36], [37].

The Middle Member comprises a 63 m thick succession of purple, massive, sandy mudstone with thin intercalations of white, coarse to medium-grained sandstone. The sandstone beds usually comprise individual tabular bodies less than 20 cm thick, characterized by the presence of horizontal stratification. Some of these sandstone bodies occasionally reach 1 m thick, with development of tabular cross-stratification and low angle cross-stratification. This sequence is interpreted as flood-plain deposits associated with sheet-flood and ephemeral channel deposits [38].

The Upper Member is composed of a 57.5 m thick succession of greenish gray, massive to laminated, bioturbated, slightly tuffaceous claystones. Tuff and limestone beds, 20 to 60 cm thick, are interbedded in the lower part of the Upper Member. The tuffaceous beds dominate the top of the section, with occasional conglomerates of volcanic clasts and tuffaceous matrix. This sequence is interpreted as lacustrine deposits (cf. [37]), associated with pyroclastic (ash fall) and debris flow deposits. Dinosaur remains were recovered from this member, situated 137 m from the base of the formation.

### Systematic Paleontology

Dinosauria Owen, 1842 [39]

Saurischia Seeley, 1887 [40]

Sauropodomorpha Huene, 1932 [41]

*Leonerasaurus taquetrensis* gen. et sp. nov.

urn:lsid:zoobank.org:act:CC75DDAC-0541-4C87-9F25-26EB21E64D1B

urn:lsid:zoobank.org:act:DF26F71F-0178-4C14-B4E6-C743B1A6FEA9

#### Holotype

MPEF-PV 1663 (Museo Paleontológico Egidio Feruglio, Trelew, Argentina); Anterior region of right dentary and isolated teeth, articulated series of cervical and anterior dorsal vertebrae, partially articulated posterior dorsal vertebrae, and articulated sacrum (preserved in natural contact with both ilia), right scapula and humerus, left and right ilia, right ischium, partially preserved femur, articulated metatarsal I and II, and pedal ungual. All vertebrae, the scapula, humerus, and pelvis were found in natural position, as a partially articulated specimen. The dentary, teeth, femur, and pedal remains were found within a radius of one meter from the center of the articulated specimen. No other remains were found at this site and therefore we interpret all these elements as belonging to a single individual.

#### Etymology

*Leoneras*, in reference to the lithostratigraphic unit where this taxon was found; *saurus*, lizard (Latinized Greek). The species name *taquetrensis* refers to the Sierras de Taquetrén, where Las Leoneras Formation crops out in Central Patagonia.

#### Locality and Age

Cañadón Las Leoneras, south of Cañadón del Zaino (both of which are affluent of the left margin of the Chubut river), southeast of Sierra de Taquetrén, Chubut Province, Central Patagonia, Argentina (Figure 1). Precise locality information is deposited at the MPEF collection and can also be obtained from the first author upon request.

The specimen was found approximately 42 m below the top of the Leoneras Formation [28], a unit considered as Lower Jurassic in age by Nakayama [28], and more specifically referred to the Pliensbachian–Toarcian [32] or Upper Sinemurian–Toarcian [33], although no direct datings of these sediments are available. The age of the Las Leoneras Formation is certainly constrained by the Middle Jurassic dating of the volcanic facies of the overlying Lonco Trapial Formation [31], [34], [35]. Furthermore, the base of the Lonco Trapial Formation in this region contains sedimentary facies with a well preserved taphoflora that was originally regarded as Middle Jurassic in age [29]–[30], although new evidence suggests this taphoflora is Early Jurassic in age [42], based on comparisons with the flora from the Early Jurassic of northwestern Patagonia and the Antarctic peninsula.

Figari and Curtade [32] interpreted the sequence of Las Leoneras Formation as initial rifting deposits, linked to the genesis of the Cañadón Asfalto Basin. It must be noted that similar rifting deposits of other regions of Patagonia have been linked to the initial break-up of southeastern Gondwana, in which small and narrow depocenters were formed by continental extension and strike-slip movements during the Upper Triassic and Lower Jurassic [43]–[44]. Therefore, the geological context of the area and the stratigraphic relationship with the Lonco Trapial Formation are consistent with a Lower Jurassic age for the Las Leoneras Formation. However, an Upper Triassic age cannot be completely ruled out at the moment, as there is not a well-defined lower constraint for the age of this unit.

#### Diagnosis

*Leonerasaurus* is a small basal sauropodomorph diagnosed by a unique combination of characters including the following autapomorphies: anterior teeth with low, spoon-shaped crowns (SI = 1.3); dorsosacral rib attached to preacetabular process of ilium (paralleled in *Lufengosaurus*); neural arches of primordial sacrals positioned on the anterior half of the centrum; caudosacral rib directed anterolaterally; humeral deltopectoral crest low and medially deflected along its distal half; flattened ischial shafts (paralleled in *Anchisaurus*). *Leonerasaurus* differs from most basal sauropodomorphs in the presence of the following characters: straight anterior region of the dentary; slightly procumbent teeth without marginal denticles and with convex labial surface and concave lingual surface; four sacral vertebrae, with two primordial sacrals bounded by a dorsosacral and a caudosacral; preacetabular process of ilium exceeding pubic peduncle and dorsoventrally low (except for *Anchisaurus* and *Mussaurus*). Finally, several plesiomorphic features distinguish *Leonerasaurus* from basal sauropods: teeth lacking labial or lingual grooves; posterior teeth with large denticles oriented at 45 degrees from tooth's margin and slightly developed wrinkling pattern; vertebral centra amphicoelous and acamerate; cervical vertebrae low and moderately elongated, without postzygodiapophyseal lamina, with elongated prezygapophyses; dorsal vertebrae with low neural arches and neural spines elliptical in cross section; absence of spinoprezygapophyseal laminae in all dorsals and of prezygodiapophyseal lamina in mid-dorsals; posterior dorsals with dorsoventrally low hyposphene-hypantrum; proximal metatarsal II hour-glass shaped in proximal view.

### Description

#### Dentary and teeth

**Figure 3:**
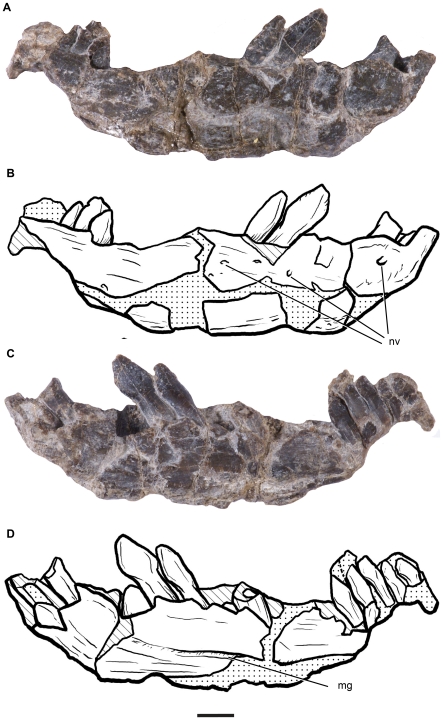
Dentary of *Leonerasaurus taquetrensis* (MPEF-PV 1663). A-B, lateral view. C–D, medial view. Scale bar represents 5 mm. *Abbreviations*: *de*, dentary; *mg*, meckelian groove; *nv*, neurovascular foramina.

The anterior region of the right dentary is the only craniomandibular element preserved in MPEF-PV 1663 (Figure 3). This element is poorly preserved but some details of its anatomy can be observed. The anterior (symphyseal) region is straight and only gently arched medially, as in non-eusauropod sauropodomorphs, contrasting with the medially broadly arched symphyseal region and anterior portion of the tooth row of basal eusauropods [45]. Although the ventral edge of the dentary has not been perfectly preserved, it does not appear to be ventrally deflected at the symphysis as in some basal sauropodomorphs (e.g., *Plateosaurus engelhardti*; [46]). The lateral surface of the dentary is flat and pierced by several neurovascular foramina (Figure 3). The longitudinal ridge that characterizes some basal sauropodomorphs (e.g., *Massospondylus carinatus*, *Coloradisaurus brevis, Plateosaurus engelhardti*; [47]) is absent from the lateral surface of the dentary, although this structure is located towards the posterior end of the tooth row and may have not been preserved in MPEF-PV 1663. The Meckelian groove is exposed on the medial surface of the dentary (close to its ventral margin), as the splenial has not been preserved in this specimen. Although the labial alveolar edge seems to be slightly more dorsally located than the lingual edge, *Leonerasaurus* does not seem to have the well-developed lateral plate that covers the labial base of the tooth crowns in eusauropods and its closest relatives [48].

**Figure 4:**
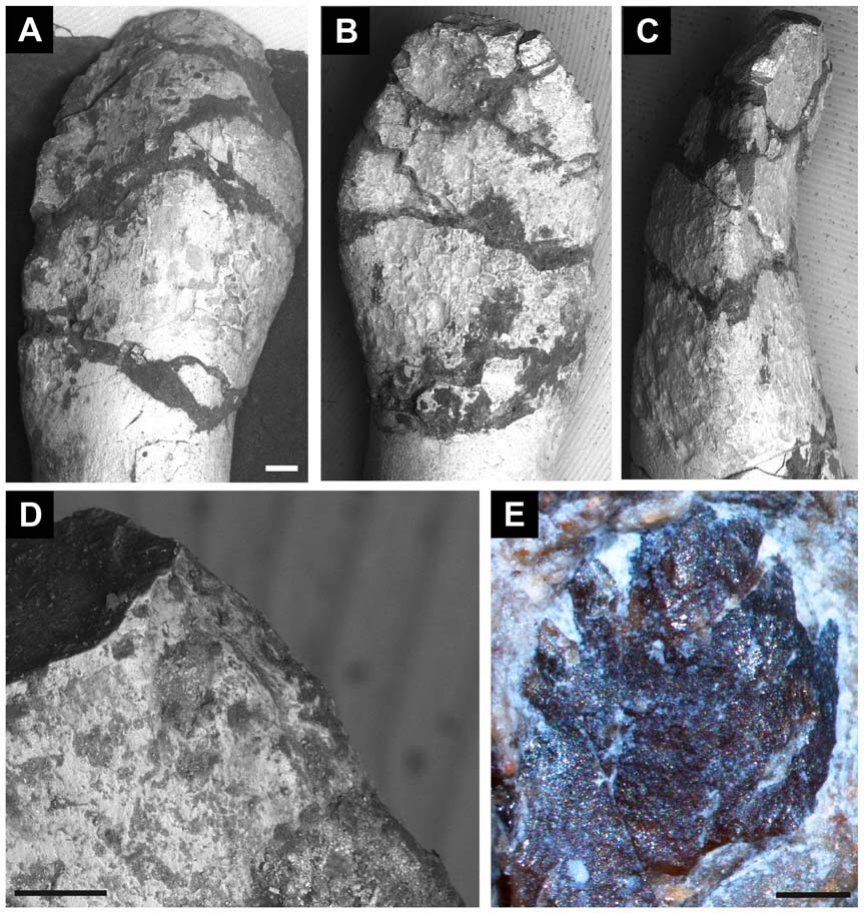
Teeth of *Leonerasaurus taquetrensis* (MPEF-PV 1663). A–D, SEM image of anterior tooth in A, labial; B, lingual; and C, mesial views. D, detail of unserrated apical region of mesial margin. E, posterior replacement tooth with denticles in lingual view. Scale bars represent 500 µm (A–C, E) and100 µm (D).

There are 13 teeth (or tooth fragments) and two empty alveoli preserved in the dentary of MPEF-PV 1663, yielding a tooth-count of 15 teeth for *Leonerasaurus* (a minimum bound given that the posterior end is broken and some alveoli may have not been preserved). Additionally, three isolated teeth of this taxon have been found in the matrix surrounding the mandibular remains (Figure 4). The teeth are slightly procumbent, forming an angle of 60 degrees with the longitudinal axis of the dentary (Figure 3), a condition found in eusauropods and in the juvenile specimens of *Mussaurus patagonicus* [49]. The crowns of dentary teeth of *Leonerasaurus* are lanceolate and separated from the root by a marked constriction. As in all basal sauropodomorphs (except for *Yunnanosaurus huangi*; [50]), the crowns of adjacent teeth are in contact and overlap each other, with the distal margin covering labially the mesial edge of the following element. Overlapping facets, however, are not present in the isolated teeth. The tooth crowns decrease in size posteriorly, with the anterior crowns higher and mesiodistally wider than the posterior ones. Based on this trend the two isolated teeth are interpreted as belonging to the anterior portion of the tooth row, as their maximum mesiodistal width is similar to that of the fourth and fifth dentary teeth (ranging between 4.5–4.9 mm).

The mesial and distal margins are asymmetrical in all preserved teeth. The mesial edge is more convex and reaches its widest point at the mid-height of the crown, whereas the convexity of the distal edge is much more gently developed and is more prominent close to the base of the crown (Figure 4). All anterior teeth lack denticles on the margins of the crown, in contrast to most non-neosauropod sauropodomorphs. Although parts of these margins are broken, one of the isolated teeth shows that the mesial and distal edges are smooth (Figure 4). The presence of small serrations at the crown's apex cannot be ruled out, as this portion is damaged in most teeth. However, if present, the denticles would be restricted to the apical tip of the crown, as in *Mussaurus patagonicus* [49], *Yunnanosaurus huangi* [51], and *Lamplughsaura dharmaramensis* [52] but in contrast with the more extensive denticulation of other non-eusauropod sauropodomorphs. The margins of most posterior teeth are damaged, but an unerupted element in the dentary bears large denticles oriented at approximately 45 degrees from the tooth's margin, resembling those of most basal sauropodomorphs (Figure 4). This pattern of anterior teeth with smooth margins and posterior teeth with lower crowns with well-developed denticles resembles the condition of the juvenile specimens of *Mussaurus patagonicus* [49], *Melanorosaurus readi* [12], and *Lamplughsaura dharmaramensis* [52] among non-sauropod sauropodomorphs. The crown's margins of *Leonerasaurus* lack the high-angled wear facets that characterize eusauropod teeth [18].

The labial surface of the tooth crowns is markedly convex both apicobasally and mesiodistally, whereas the lingual surface is concave in the anteriormost elements, resulting in a spoon-shaped crown (Figure 4). The concave lingual surface of some teeth in *Leonerasaurus* is not as developed as in Eusauropoda, although an incipient condition has also been noted for some basal sauropods (e.g., *Chinshakiangosaurus chunghoensis* [48], *Tazoudasaurus naimi* [53]) and *Lamplughsaura dharmaramensis* [52]. The crowns of *Leonerasaurus*, however, lack distinct grooves in their labial or lingual surfaces, which occur in eusauropods and some basal sauropods [48]. The enamel outer surface has been damaged in some teeth, but in at least some posterior teeth the base of the crown bears regions of wrinkled enamel (Figure 4). This texture is much more faintly developed than the coarse wrinkling synapomorphic of Eusauropoda [18], as in other basal sauropods and other sauropodomorph taxa (e.g., *Anchisaurus polyzelus*, *Mussaurus patagonicus*, *Melanorosaurus readi,* and *Lamplughsaura dharmaramensis*).

#### Cervical vertebrae

**Figure 5:**
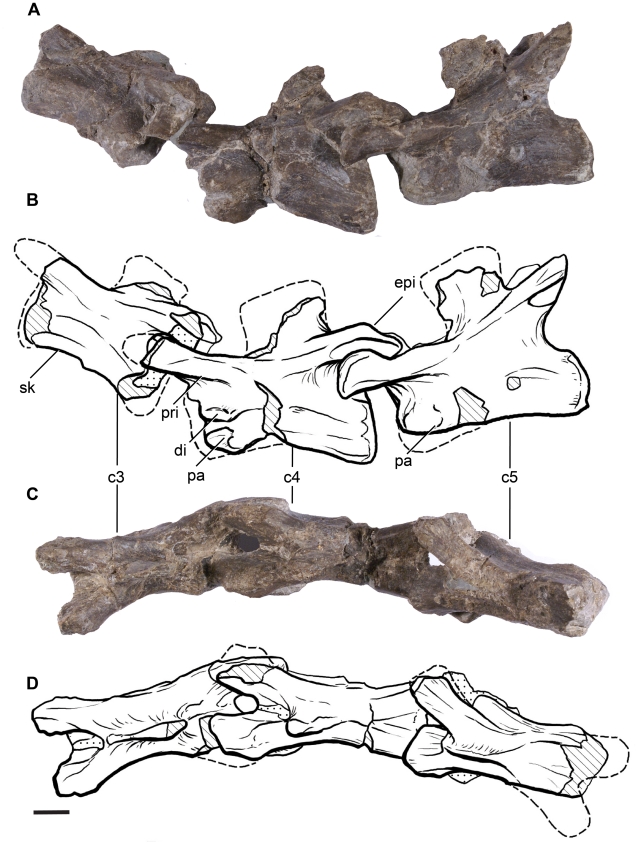
Cervical vertebrae 3–5 of *Leonerasaurus taquetrensis* (MPEF-PV 1663). A–B, lateral view. C–D, dorsal views. Scale bars represent 10 mm. Hatched pattern represents broken surfaces and dotted pattern represents sediment. *Abbreviations*: *c3-c5*, cervical vertebrae 3 through 5; *di*, diapophysis; *pa*, parapophysis; *pri*, prezygapophyseal ridge; *epi*, epipophysis; *psf*, postspinal fossa; *sk*, sagittal keel.

**Figure 6:**
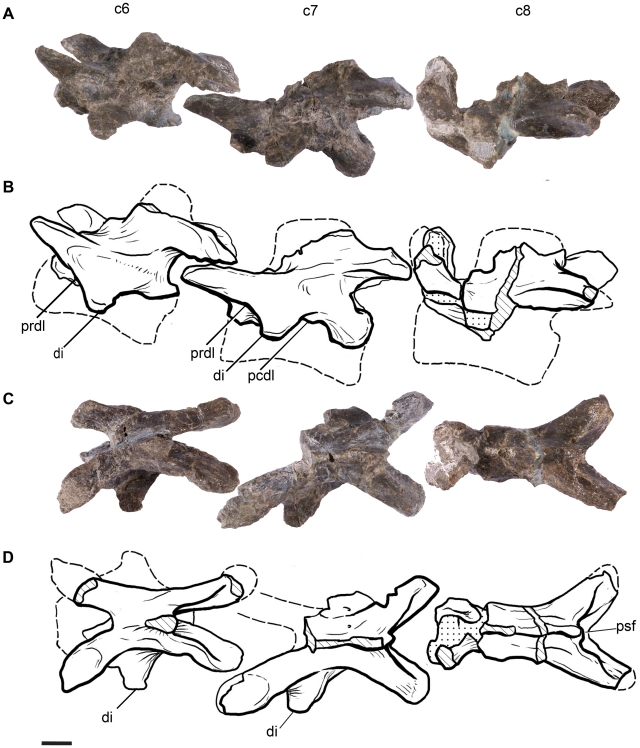
Cervical vertebrae 6–8 of *Leonerasaurus taquetrensis* (MPEF-PV 1663). A–B, lateral view. C–D, dorsal views. Scale bars represent 10 mm. Hatched pattern represents broken surfaces and dotted pattern represents sediment. *Abbreviations*: *c6-c8*, cervical vertebrae 6 through 8; *di*, diapophysis; *pcdl*, posterior centrodiapophyseal lamina; *prdl*, prezygodiapophyseal lamina; *psf*, postspinal fossa.

Nine cervical vertebrae are preserved in MPEF-PV 1633, including the axis and the eight subsequent elements preserved in two sections of articulated vertebrae (Figures 5, 6). Although the atlas has not been preserved, *Leonerasaurus* would have ten cervical vertebrae with this missing element, as in other non-eusauropod sauropodomorphs. The axis is poorly preserved, but the centrum is relatively short with respect to its dorsoventral height (as in *Melanorosaurus readi* [12]; see Table S1 for measurements). The postzygapophyses project marginally beyond the posterior end of the axial centrum.

The anterior cervical vertebrae of *Leonerasaurus* are low and moderately elongated (see Table S1 for measurements) as in most basal sauropodomorphs, with the height of the neural arch less than that of the centrum (Figure 5). Basal sauropods (including *Lessemsaurus* and *Tazoudasaurus* [53]), instead, have much higher cervical neural arches with depressions on their anterior and posterior surfaces [7], [12], [50]. The neural arches of these anterior cervicals are fused to the centra, and the neurocentral suture is completely closed, suggesting MPEF-PV 1663 is not a juvenile individual (see below). The neural spines of most cervicals are damaged, except for the spine of the fifth vertebra. This neural spine is not slanted anteriorly and is approximately as long as high, resembling the condition of sauropods and closely related taxa (e.g., *Melanorosaurus* [12]), but unlike the extremely long and low spines of more basal sauropodomorphs.

The parapophyses are small ridge-like projections located close to the anterior margin of the anterior cervicals (C3–C5). The parapophyses of more posterior cervicals have not been preserved, as the preserved centra of these vertebrae have been severely damaged. The diapophyses gradually increase their lateral projection along the cervical series, are located well below the postzygapophysis and lack a postzygodiapophyseal lamina. The latter lamina is absent in most basal sauropodomorphs (including basal sauropods such as *Lessemsaurus*; [7]) and is only present in *Tazoudasaurus* and eusauropods [53]. The diapophyseal laminae are poorly developed in all cervicals, although the posterior cervicals have a moderate development of the posterior centrodiapophyseal lamina and the prezygodiapophyseal laminae, as in most non-sauropod sauropodomorphs. The development of these laminae, however, does not reach the degree of development present in cervicals of *Tazoudasaurus* and more derived sauropodomorphs.

The prezygapophyses are elongated, being approximately 50% the entire length of the neural arch. The prezygapophyses of the anterior cervicals extend horizontally, whereas those of more posterior cervicals are slightly upturned. The lateral surface of all cervical prezygapophyes bears a longitudinal ridge that extends close to their anterior edge (Figures 5, 6). This ridge can be interpreted as an incipient lamina, given that it is continuous with the prezygodiapophyseal lamina in posterior cervicals. In anterior vertebrae (C3–C5) a well-developed lamina is absent but the ridge is nonetheless present.

The cervical postzygapophyses bear on their dorsal surface epipophyses, although most of them were damaged during preservation. Along the cervical series, the postzygapophyses gradually increase in size and change their orientation. The most anterior postzygapophyses are directed posterodorsally, with their major axis forming an angle of approximately 30 degrees with the horizontal. These postzygapophyses are relatively small and lack well developed spinopostzygapophyseal laminae and postspinal fossae between them. The postzygapophyses of the last cervicals, instead, are directed sub-parallel to the horizontal axis and are much larger with respect to the anteroposterior length of the neural arch. In these posterior elements, the postzygapophyses bear well developed spinopostzygapophyseal laminae that bound a deep postspinal fossa (Figure 6).

All the preserved cervical centra are acamerate and amphicoelous, as in all sauropodomorphs more basal than *Tazoudasaurus* and Eusauropoda [45], [53]. The articular surfaces of the centra are subequal in height and width, as in all basal sauropodomorphs. The length/height ratio of the best preserved cervical centra of *Leonerasaurus* (C3–C5) is approximately 3.2, resembling the condition of most basal sauropodomorphs, except for the long-necked massospondylids (*Massospondylus*, *Coloradisaurus*, *Lufengosaurus*) and some derived groups of eusauropods (e.g., *Omeisaurus* [54], *Mamenchisaurus*). The anterior cervical centra are only slightly constricted at their midpoint but are markedly constricted at the cervicodorsal transition, the centra having a minimum width that is 62% of the width of the posterior articular surface. All cervical vertebrae bear a noticeable sagittal keel running on the ventral surface of the centra.

#### Dorsal vertebrae

**Figure 7:**
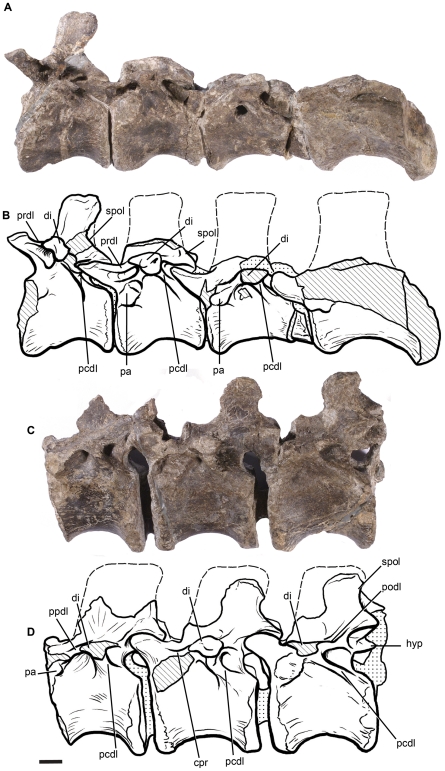
Dorsal vertebrae of *Leonerasaurus taquetrensis* (MPEF-PV 1663) in lateral view. A–B, first four dorsals. C–D, mid-posterior dorsals. Scale bar represents 10 mm. Hatched pattern represents broken surfaces and dotted pattern represents sediment. *Abbreviations*: *cpr*, centroprezygapophyseal ridge; *di*, diapophysis; *hyp*, hyposphene; *pa*, parapophysis; *pcdl*, posterior centrodiapophyseal lamina; *podl*, postzygodiapophyseal lamina; *ppdl*, parapodiapophyseal lamina; *prdl*, prezygoodiapophyseal lamina; *spol*, spinopostzygapophyseal lamina.

The dorsal series is represented by articulated elements of the anterior and mid dorsal vertebrae. The most complete elements include the first five dorsals that have been preserved in articulation with the cervicals, a probable sixth dorsal, and a group of three articulated mid-dorsals (Figure 7). Fragments of more posterior dorsal vertebrae were scattered in the matrix, together with dorsal ribs.

All of the preserved dorsals have their neural arches fused to the centra, and the neurocentral suture is completely closed, although its trace can be distinguished in some of the mid-dorsal vertebrae. This condition also suggests MPEF-PV 1663 is not a juvenile individual (see below). The neural arches of dorsal vertebrae are relatively anteroposteriorly long and dorsoventrally low (see Table S1 for measurements), with their height ranging between 70% and 90% of the centrum height, as in non-sauropod sauropodomorphs. In *Lessemsaurus*, *Antetonitrus,* and more derived sauropodomorphs, the neural arches are higher than the centrum height. Given the low height of the neural arch pedicles, the neural canal of the dorsal vertebrae of the new taxon is subcircular rather than dorsoventrally elongated. The neural arches of *Leonerasaurus* are also plesiomorphic in having a narrow anterior surface occupied by the centroprezygapophyseal ridge, instead of having the broad concave surface present in most eusauropods and in posterior dorsals of *Lessemsaurus* [7]. The neural spines of mid to posterior dorsals of *Leonerasaurus* are low and anteroposteriorly elongated (its dorsoventral height is two thirds the length at its base), in contrast to the dorsally elongated spine of *Melanorosaurus* and sauropods [12]. In the most anterior dorsals, however, the spines are relatively higher, being 150% of the anteroposterior length of their bases. The dorsal neural spines are mediolaterally narrow and elliptical in cross section and lack spinodiapophyseal laminae, sharing the plesiomorphic condition of most basal sauropodormorphs.

The parapophyses of the most anterior dorsals are located close to the anterior edge of the vertebrae at the neurocentral suture (Figure 7), unlike the more posteriorly positioned parapophyses of *Lessemsaurus* and more derived sauropods [12]. Along the dorsal series, the parapophyses gradually shift their position posterodorsally, with the third dorsal vertebra as the first element that has the parapophysis completely located on the base of the neural arch. The parapophyses only reach the dorsoventral midpoint of the neural arch pedicles in the mid-dorsals. None of the dorsal vertebrae of *Leonerasaurus* has the anterior centroparapophyseal lamina or the prezygoparapophyseal lamina present in *Tazoudasaurus* and more derived sauropods [53]. The diapophyses (and transverse processes of posterior elements) are also plesiomorphic in being directed horizontally, as in all non-eusauropod sauropodomorphs. The dorsal diapophyses of *Leonerasaurus* are connected with the parapophyses through the anterior diapoparapophyseal laminae and with the centrum through the posterior centrodiapophyseal laminae, as in all saurischian dinosaurs. The prezygodiapophyseal lamina is present in anterior dorsals and forms the dorsal roof of a deep anterior depression. This lamina, however, is absent in the mid-dorsals, resembling the generalized condition of basal sauropodomorphs. Eusauropods and closely related taxa (e.g., *Tazoudasaurus* [53]) differ from the plesiomorphic condition by having this lamina present throughout the dorsal series.

The prezygapophyses are long and projected cranially in anterior dorsals, but become shorter and anterodorsally projected in mid-dorsals. None of the preserved dorsals of *Leonerasaurus* have the spinoprezygapophyseal laminae present in sauropods (including the incipiently developed laminae of posterior dorsals in basal forms such as *Antetonitrus* and *Lessemsaurus*). The postzygapophyses have broad and subcircular articular facets in all preserved dorsals. The dorsal surface of the postzygapophyses bears a moderately developed spinopostzygapophyseal lamina that bounds a deep postspinal fossa (as in posterior cervicals). The spinopostzygapophyseal laminae of *Leonerasaurus* are less developed than in the basal sauropods *Lessemsaurus* and *Antetonitrus*, and much less than in *Tazoudasaurus* and eusauropods. The hyposphene-hypantrum articulations are either poorly preserved or not exposed in all but the most posterior of the preserved dorsal vertebra. The dorsoventral extension of this hyposphene is approximately 70% the height of the neural canal, the generalized condition of basal sauropodomorphs. *Melanorosaurus* and more derived forms (i.e., sauropods), instead, have dorsoventrally deeper hyposphenes [12].

The centra of all preserved dorsals are amphicoelous and acamerate. Along the dorsal series the centra become proportionately shorter and higher, although all vertebrae have an elongation index above 1.0, as do all non-eusauropods [50]. The lateral surface of the dorsal centra is only slightly depressed, lacking the discrete excavaction or fossa present in basal sauropods (e.g., *Lessemsaurus*; [11]) or the pleurocoels that characterize eusauropods [18].

#### Sacrum

**Figure 8:**
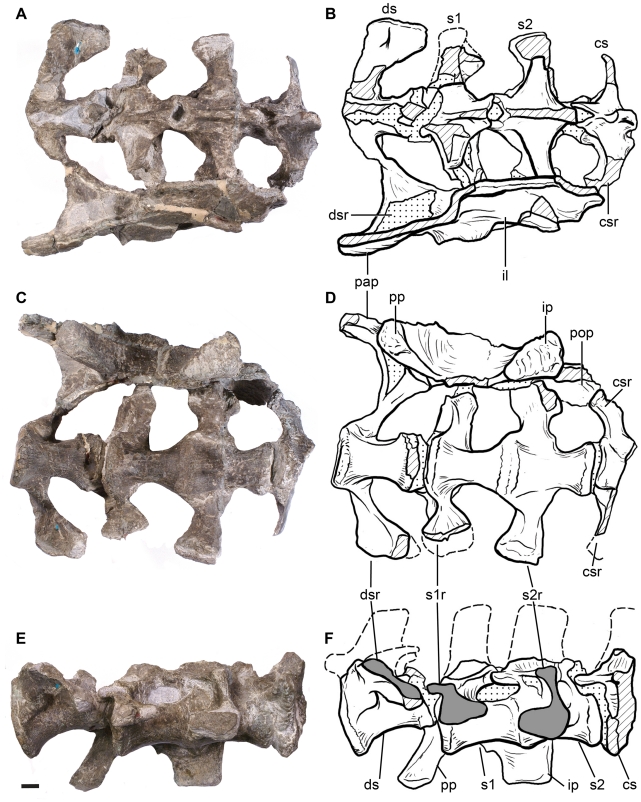
Sacral vertebrae of *Leonerasaurus taquetrensis* (MPEF-PV 1663). A–B, dorsal view. C–D, ventral view. E–F, lateral view (inverted right side). Hatched pattern represents broken surfaces and dotted pattern represents sediment. Gray areas represent the iliac attachment surface of the sacral ribs. Scale bar represents 10 mm. *Abbreviations*: *cs*, caudosacral; *csr*, caudosacral rib; *ds*, dorsosacral; *dsr*, dorsosacral rib; *il*, ilium; *ip*, ischial peduncle; *s1*, first primordial sacral; *s1r,* first primordial sacral rib; *s2r*, second primordial sacral rib; *s2r*, second primordial sacral rib; *pap*, preacetabular process; *pop*, postacetabular process; *pp*, pubic peduncle.

Four sacral vertebrae were found in natural articulation with both ilia (Figure 8). All centra have subcircular articular facets (see Table S1 for measurements), and their ventral surface is smooth and lacks either a keel or a shallow groove. All sacral ribs contact the ilium, but these are not fused to the ilium and are not distally fused among them, forming a sacricostal yoke (Figure 8E). The internal two sacral vertebrae are identified as the primordial sacrals and the anteriormost and posteriormost vertebrae are therefore identified as a dorsosacral and a caudosacral elements. The identification of the primordial sacral is based on the following criteria: fusion of sacral centra, morphology of the transverse processes and sacral ribs, and area of attachment to the ilium. The central sacral elements are the only sacrals that have fused their centra through their articular facets. This is consistent with the pattern of sacral fusion noted for sauropods, in which the two first elements that fuse together have been interpreted as the primordial sacrals [18]. The morphology of the transverse processes and sacral ribs also indicates that the internal sacral elements are the primordial sacrals. As in most basal sauropodomorphs, the first primordial sacral of *Leonerasaurus* has a particular morphology of the rib, with concave anterior and posterior surfaces that are roofed by the anteroposteriorly expanded transverse process. Similarly, the second primordial sacral has an L-shaped sacral rib, with an anterior concavity roofed by the transverse process (Figure 8F). This morphology is absent in the transverse process and sacral rib of the anteriormost and posteriormost sacral vertebrae. Furthermore, the sacral rib of the most anterior element of the sacrum resembles the dorsosacral vertebrae of other basal sauropodomorphs in being anteroposteriorly long, obliquely oriented, and attaching to the preacetabular region of the ilium (e.g., *Lufengosaurus*).

The most anterior sacral vertebra (dorsosacral) is located between the anterior end of the preacetabular process and the pubic peduncle of the ilium. Its neural arch is low, and most of its dorsal surface is damaged. The centrum is well constricted at its midpoint. The transverse process is fused to the sacral rib, forming a single complex that extensively contacts the ilium. The origin of the transverse process on the lateral surface of the centrum is long and occupies approximately 47% of the anteroposterior length of the centrum (Figure 8C). The sacral rib markedly expands towards the ilium as a flat lamina that extends obliquely in an anterodorsal-posteroventral direction. The elongated and obliquely oriented articular surface of the dorsosacral rib resembles that of some basal sauropodomorphs (e.g., *Riojasaurus*, *Lufengosaurus*, *Melanorosaurus*) but is unlike the rounded iliac articulation of the dorsosacral rib of other taxa (e.g., *Anchisaurus* YPM 208, *Massospondylus*). The anterodorsal area of attachment occupies the medial surface of the preacetabular process, as in *Lufengosaurus huenei*. Other sauropodomorphs, however, have the anterior area of attachment of the dorsosacral rib located more ventrally, on the pubic peduncle (e.g., *Melanorosaurus readi*). The posterodorsal surface of the laminar dorsosacral rib is flat and the anteroventral surface bears a slight concavity bounded ventrally by a thick ventral margin of the rib.

The subsequent sacral vertebra (first primordial sacral) is located at the level of the anteroposterior center of the acetabulum. The centrum is more constricted at its midpoint than in the other sacral centra, and its neural arch is anteroposteriorly shorter than those of the other sacrals. The pedicles of the neural arch are shifted anteriorly, extending only along the anterior half of the centra (Figure 8E), as in the first primordial sacral of *Yunnanosaurus huangi*. This neural arch has preserved the base of a mediolaterally narrow neural spine that extends along the entire dorsal surface of the neural arch. The transverse process originates on the neural arch as an anteroposteriorly broad horizontal lamina, which tapers rapidly along its lateral projection and ends in a narrow tip (Figure 8A), as in the primordial sacral of most basal sauropodomorphs (*Thecodontosaurus* YPM 2192, *Efraasia* SMNS 14881, *Plateosaurus*, *Riojasaurus*, *Melanorosaurus*). The transverse process and the sacral rib are fused to each other, but we interpret this constriction as the lateral end of the transverse process. The sacral rib is L-shaped and is formed by a high vertical lamina with a thin dorsal edge and a more robust horizontal lamina that projects posteriorly and is located ventrally, at the level of acetabular roof (Figure 8E). Thus, the sacral rib has a deep concavity that faces posteriorly and is partially roofed by the anteroposteriorly broad transverse process. Given its anteroposterior breadth, the transverse process also extends anteriorly from the vertical lamina of the rib, creating a slightly concave anterior surface of the transverse process-sacral rib complex. This particular morphology of the rib with an anterior and posterior concavity roofed by the transverse process is also present in the first primordial sacral of most sauropodomorphs (*Riojasaurus*, *Melanorosaurus*). However, in some of the most basal taxa of this clade the transverse process does not anteriorly overhang the sacral rib, and therefore the anterior concavity is not present (*Saturnalia* [55], *Thecodontosaurus* YPM 2192, *Efraasia* SMNS 14881, *Plateosaurus*). In ventral view, the medial area of attachment of the complex is anteroposteriorly broad and occupies the anterior half of the centrum. The lateral contact with the ilium is only moderately expanded anteroposteriorly (Figure 8C).

The third vertebra of the sacrum (second primordial sacral) is located at the level of the ischial peduncles of the ilium. The centrum is broader and less constricted at its midpoint than other sacral centra. Its neural arch is relatively long and also placed anteriorly on the centrum (Figure 8E). The neural spine is narrow and anteroposteriorly extensive and occupies the entire dorsal surface of the neural arch. The spine projects anteriorly together with the prezygapophyses, exceeding the anterior margin of the pedicles of the neural arch and the vertebral centrum. The transverse process originates from the anterior half of the vertebra and projects posterolaterally. The anteroposterior extension of the dorsal surface of the transverse process tapers only mildly along its medial half, and then it maintains a constant breadth. As in the previous vertebra, the sacral rib is L-shaped, with a thin vertical lamina that extends from the posterior margin of the transverse process to a horizontal process that is ventrally located and dorsoventrally thick (Figure 8E). In this vertebra, however, the horizontal process extends anteriorly, creating a deep cranially facing concavity roofed by the relatively broad transverse process. Such morphology closely resembles the second primordial sacral of basal sauropodomorphs (*Saturnalia* [55], *Thecodontosaurus* YPM 2192, *Efraasia* SMNS 14881, *Plateosaurus*, *Riojasaurus*), although in some taxa the roof of the transverse process is highly reduced (*Yunnanosaurus*, *Melanorosaurus* NM QR1551). The area of attachment of the rib with the centrum is more extensive than in other vertebrae and occupies up to 60% of the ventral surface of the centrum (Figure 8C). In ventral view, the rib is hour-glass shaped, with a central constriction and a lateral marked expansion towards the contact with the ilium.

Finally, the most posterior element of the sacrum (caudosacral) is only partially preserved. Most of the centrum is missing, except for the area of attachment of the left sacral rib. The base of the neural spine of this vertebra is approximately twice as broad as those of the preceding elements and is united to the prezygapophyses by an incipiently developed spinoprezygapophyseal lamina. As in the previous vertebrae, the dorsal surface of the transverse process is anteroposteriorly broad and tapers laterally. On the ventral surface, the sacral rib has an anteroposteriorly short attachment to the vertebral centrum. The rib projects anterolaterally from the anterior edge of the centrum, gradually broadening towards the postacetabular process of the ilium (Figure 8C). This expansion is partially roofed by the horizontal lamina of the transverse process, forming an anteriorly facing concavity. The contact of this rib with the ilium has not been preserved because the postacetabular process is not complete, but the broad lateral end of this process indicates this vertebra was firmly sutured to the ilium. The presence of a caudosacral vertebra is an uncommon feature among basal sauropodomorphs (see Discussion). The caudosacral rib of *Leonerasaurus* is rather different from the caudosacral of *Plateosaurus* that is directed posterolaterally and greatly expanded towards its lateral ends [56]–[57].

#### Pectoral girdle

**Figure 9:**
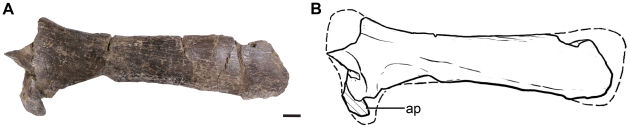
Right scapula of *Leonerasaurus taquetrensis* (MPEF-PV 1663) in lateral view. **A**, photograph; **B**, interpretive line drawing. Scale bar represents 10 mm. Hatched pattern represents broken surfaces. *Abbreviations*: *ap*, acromion process.

The right scapula is the only preserved element of the pectoral girdle. The scapula of *Leonerasaurus* has the generalized morphology of basal sauropodomorphs. The dorsal blade is poorly expanded (Figure 9), with an anteroposterior extension that comprises 22% of the total length of the scapula (as preserved; see Table S1 for measurements). Although the dorsal margin of the scapular dorsal blade is poorly preserved, it is unlikely that *Leonerasaurus* had the abrupt and marked expansion present in some sauropodomorphs. The scapular shaft has almost straight edges and is elongated and narrow, as its minimum anteroposterior width is approximately 15% the total scapular dorsoventral length. This falls within the range of most basal sauropodomorphs. This ratio could actually be smaller in *Leonerasaurus*, because the ventral end is incomplete. Basal sauropods (*Lessemsaurus*, *Antetonitrus*, *Vulcanodon*) and the closely related *Melanorosaurus* (NM QR1551) have a much broader scapula, a condition that was subsequently reverted in eusauropods [12], [50]. The ventral end of the scapula is not complete, although it can be determined that the acromial process formed an angle of approximately 45 degrees with the dorsoventral axis of the scapula, as in non-eusauropod sauropodomorphs (with the exception of *Saturnalia tupiniquim*, *Coloradisaurus brevis*, *Lufengosaurus huenei*, *Massospondylus carinatus*). Although the anteroposterior extension of the acromion process cannot be determined for *Leonerasaurus*, it is likely that this process was relatively short as in non-eusauropod sauropodomorphs. Posterior to the acromial process, the lateral surface of the scapula has a shallow and poorly delimited concavity.

#### Forelimb

**Figure 10:**
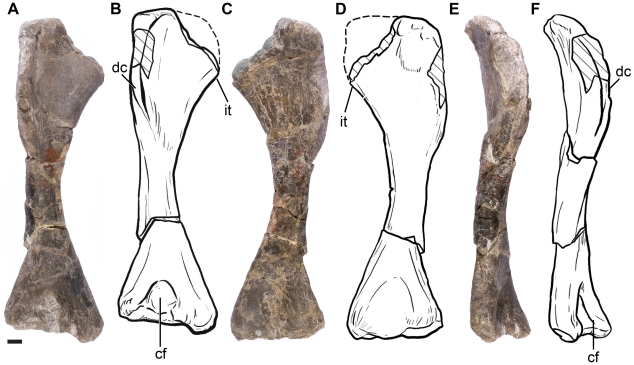
Right humerus of *Leonerasaurus taquetrensis* (MPEF-PV 1663). A–B, anterior view. C–D, posterior view. E–F, lateral view. Scale bar represents 10 mm. Hatched pattern represents broken surfaces. *Abbreviations*: *cf*, cuboid fossa; *dc*, deltopectoral crest; *it*, internal tuberosity.

The right humerus is the only element of the forelimb preserved in the holotype of *Leonerasaurus* (see Table S1 for measurements). The humerus is gracile and is more expanded distally than proximally (Figure 10). The lateromedial expansion of the proximal end is moderately well developed (approximately 28% the total humeral length as preserved) and probably resembled the condition of other non-eusauropod sauropodomorphs (except for massospondylids). The proximal articular surface has not been preserved, but the proximomedial region is expanded, marking the origin of the internal tuberosity. The incompleteness of this region, however, precludes determining if the internal tuberosity of *Leonerasaurus* was as developed as in *Massospondylus* (and related forms), or moderately developed as in other basal sauropodormophs (e.g., *Saturnalia*, *Melanorosaurus* [10]).

The deltopectoral crest rises gradually from the proximolateral edge of the humerus and extends distally for approximately 50% of the humeral length (Figure 10). This is the generalized condition of adult non-sauropod sauropodomorphs (including *Melanorosaurus*; [10]), whereas in sauropods the crest usually has a more restricted extension (even in basal forms such as *Antetonitrus*, *Lessemsaurus*, *Vulcanodon*). The deltopectoral crest, however, is low and has a rounded profile in lateral view (Figure 10E). This contrasts with the condition of most basal sauropodomorphs (*Saturnalia*, *Plateosaurus*, *Massospondylus*, *Coloradisaurus*) in which the crest is high, sharp-edged, and has a straight (vertically-oriented) profile in lateral view. The low deltopectoral crest of *Leonerasaurus* resembles that of basal sauropods (e.g., *Lessemsaurus*) and the closely related *Melanorosaurus* [10]. However, as in these taxa, the crest of *Leonerasaurus* is not as low and reduced as in eusauropods. In anterior view, the deltopectoral crest of *Leonerasaurus* runs parallel to the proximodistal axis of the humerus along its proximal half, being perpendicular to the transverse axis of the distal humeral condyles. Its distal half, however, deflects medially, forming an angle of approximately 75 degrees with the transverse axis (Figure 10A).

The humeral diaphysis is relatively long and occupies over 30% of the humeral length, giving the humerus a gracile aspect. The shaft is ovoid-shaped in cross section, with its major axis oriented mediolaterally. At the distal end, the humerus expands markedly along the lateromedial axis but is only moderately expanded in anteroposterior direction. The lateromedial expansion is approximately 35% of the total humeral length, as in most basal sauropodomorphs except for *Coloradisaurus* and *Yunnanosaurus*, which have a more expanded distal end. Eusauropods (and *Anchisaurus*) have a different condition, with only a moderately developed distal humeral expansion of less than 30% the humeral length. The anterior surface of the distal end bears a deep and well-defined and circular cuboid fossa (Figure 10A), as in the humerus of most non-eusauropod sauropodomorphs (*Plateosaurus engelhardti* MB skelett 25; *Massospondylus carinatus* SAM-PK-K391; *Lessemsaurus sauropoides*). The olecranon fossa of the posterior surface of the distal humerus is extremely shallow.

#### Pelvic girdle

**Figure 11:**
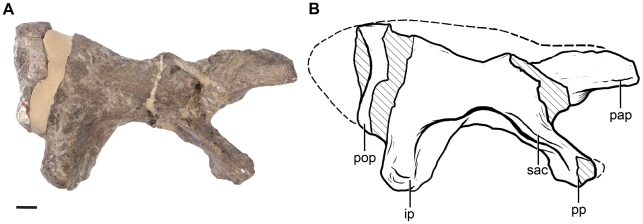
Right ilium of *Leonerasaurus taquetrensis* (MPEF-PV 1663) in lateral view. **A**, photograph; **B**, interpretive line drawing. Scale bar represents 10 mm. Hatched pattern represents broken surfaces. *Abbreviations*: *ip*, ischial peduncle; *pap,* preacetabular process; *pop*, postacetabular process; *pp*, pubic peduncle; *sac*, supracetabular crest.

The type specimen of *Leonerasaurus* includes the ilia, left ischium, and the distal part of the left pubic blade (see Table S1 for measurements). The left and right ilia were preserved in natural articulation with (but not fused to) the sacrum. The ilium of *Leonerasaurus* shares multiple features with basal sauropodomorphs that distinguish this element from the characteristic morphology of the ilium in Eusauropoda. The preacetabular process is triangular and dorsoventrally low with respect to the iliac blade above the acetabulum (Figure 11), as in most non-eusauropod sauropodomorphs. This process is remarkably extended anteriorly, slightly exceeding the cranial margin of the pubic peduncle and being slightly more than twice as long as deep. A similarly long, low, and extensive preacetabular process is only present in *Anchisaurus polyzelus* (YPM 208) and one of the specimens referred to *Melanorosaurus readi* (NM QR 3314) among sauropodomorphs. *Kotasaurus* [58] and eusauropods (e.g., *Shunosaurus*, *Barapasaurus, Patagosaurus*) also have anteriorly extensive preacetabular processes [12], [50], although these taxa have more extensive dorsoventral development of this process. Much of the dorsal blades of the ilia are not preserved, but the blade of the left ilium is relatively low at the level of the ischial peduncle as in basal sauropodomorphs. The acetabulum is mediolaterally narrow and lacks a medial wall as in all sauropodomorphs, except for the most basal forms (e.g., *Saturnalia*, *Thecodontosaurus* [12], [50], [55]. The pubic peduncle is long and subtriangular in cross section, with an acute lateral margin formed by an anteriorly located supracetabular crest (Figure 11), resembling the condition in other basal sauropodomorphs. The ischial peduncle is subequal in length but much more robust than the pubic peduncle, as in non-eusauropod sauropodomorphs. This peduncle also lacks the posterior heel present in several basal sauropodomorphs (e.g., *Plateosaurus*, *Riojasaurus*, *Coloradisaurus*). Much of the postacetabular process has not been preserved, but based on the extension of the caudosacral rib, the development of this process probably was well developed as in non-eusauropod sauropodomorphs.

**Figure 12:**
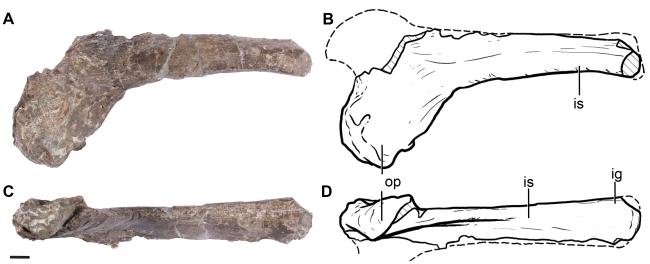
Right ischium of *Leonerasaurus taquetrensis* (MPEF-PV 1663). A–B, lateral view. C–D, posterodorsal view. Scale bar represents 10 mm. Hatched pattern represents broken surfaces. *Abbreviations*: *ig*, ischial groove; *is*, ischial shaft; *op,* obturator process.

The left ischium has the characteristic broad proximal obturator plate and narrow ischial shaft of Dinosauria (Figure 12). At the proximal plate, the robust area that articulates with the ilium is preserved, but most of the anterior extension that contacts the pubis is incomplete. The ischial shaft is not complete and lacks the distal end. The preserved portion of the shaft is a flattened lamina, teardrop shaped in cross section, with a broader external margin and a thin internal symphyseal edge (Figure 12C). In most sauropodomorphs, the ischial shaft is more robust and subcircular or subtriangular in cross section, but a flattened ischial shaft has been described for *Anchisaurus*, *Saturnalia*, and *Thecodontosaurus* [59] among basal sauropodomorphs. The plane of the laminar shaft is twisted with respect to the plane of expansion of the proximal ischium (Figure 12), forming an angle of approximately 40 degrees. Therefore, the flat surface of the ischial shaft must have faced ventrolaterally, differing from the coplanar ischial shaft of *Anchisaurus* [59]. A shallow groove runs along the dorsal margin of the entire ischial shaft, as in most sauropodomorphs. The distal end of the ischium is not present, but the distalmost preserved region is more robust and less flattened than the proximal half of the ischial shaft.

The distal region of the pubic apron preserved in MPEF-PV 1663 shows that the pubes were flat and mediolaterally broad, as in basal sauropodomorphs. The lateral margin, however, is straight rather than concave, as in massospondylids and eusauropods. The distal end of the pubis has a slightly developed boot that is almost twice the anteroposterior thickness of the flat pubic apron.

#### Hindlimb

Only the diaphysis of the femur, metatarsals I and II, and a pedal ungual have been preserved of the hindlimb. The only anatomical information that can be gathered from the fragments of the femur is that the shaft was subcircular in cross section, as in sauropodomorphs more basal than *Melanorosaurus* [12].

**Figure 13:**
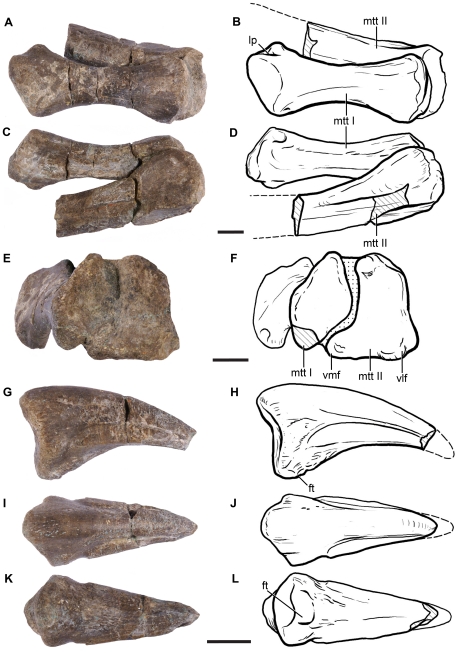
Pedal remains of *Leonerasaurus taquetrensis* (MPEF-PV 1663). A–D, metatarsal I and II in A–B, dorsal; C–D, plantar; and E–F proximal views. G–L, pedal phalanx in G–H, lateral; I–J, dorsal; and K–L, plantar views. Scale bars represent 10 mm. Hatched pattern represents broken surfaces and dotted pattern represents sediment. *Abbreviations*: *mtt I-II*, metatarsal I-II; *ft*, flexor tubercle; *lp*, ligament pit; *vlf,* ventrolateral flange; *vmf*, ventromedial flange.

The first metatarsal is completely preserved and is more than twice as long as wide (Figure 13; see Table S1 for measurements), resembling the condition of most basal non-sauropod sauropodomorphs. In sauropods and closely related taxa (e.g., *Melanorosaurus*, *Aardonyx*), metatarsal I is more robust, being the widest element of the metatarsus and usually having a length/width ratio smaller than 1.5 [50]. Similarly, the proximal surface of metatarsal I is ovoid and relatively small in comparison with that of metatarsal II, as in basal sauropodomorphs.

The proximal articular surface of the first metatarsal is also plesiomorphic in being perpendicular to the proximodistal axis of this bone, instead of having the obliquely oriented articular facet of eusauropods [45]. Although the proximal articular regions of metatarsal I and II are in tight contact, the shaft of metatarsal I is well separated from that of metatarsal II (Figure 13), as in all sauropodomorphs more derived than *Saturnalia*, *Pantydraco*, and *Efraasia* [60]. The distal articular region of metatarsal I is asymmetrical, with a thick and rounded lateral articular surface and a flattened medial region. The robust lateral articular facet projects more distally than the medial surface, producing an angled distal articulation, as in most sauropodomorphs more derived than *Saturnalia* [12]. Both the lateral and medial surfaces of the distal region bear a shallow ligament pit, with the medial pit delimited by a more developed sharp rim.

Only the proximal half of the second metatarsal has been preserved, and this element is still in articulation with metatarsal I. The proximal articular surface of metatarsal II has an hourglass shape in proximal view; the lateral and medial margins for the articulation of metatarsals I and III are strongly concave (Figure 13E), as in all sauropodomorphs more basal than *Vulcanodon* and eusauropods [46]. The ventrolateral flange of the proximal articular surface is small and less developed than the ventromedial flange, in contrast to the condition of most massospondylids and basal sauropods (*Antetonitrus*, *Lessemsaurus*, *Tazoudasaurus*), but similar to *Melanorosaurus* and other basal sauropodomorphs [60]. Similarly, the second metatarsal of *Leonerasaurus* is distinguished from that of massospondylids by the absence of a well-developed facet for articulation with the medial distal tarsal on the proximolateral corner of its plantar surface [60].

The only preserved pedal ungual has the characteristic shape of sauropodomorphs more basal than *Vulcanodon* and *Tazoudasaurus*. The ungual of *Leonerasaurus* is straight, pointed, moderately recurved, and has a broad flat ventral surface that is separated from the lateral and medial surfaces by a sharp ridge (Figure 13). The lateral and medial surfaces bear a deep groove that bifurcates proximally. The proximal articular surface is subtriangular in shape and is composed of two shallow and concave articular facets with a broad ventral base. The proximoventral surface of the ungual bears a small flexor tubercle. Many of these ungual characters of *Leonerasaurus* also show the plesiomorphic condition with respect to the unguals of the basal sauropods *Lessemsaurus* and *Antetonitrus*.

## Discussion

### Phylogenetic position

The phylogenetic affinities of *Leonerasaurus* were tested through a cladistic analysis within the context of basal Sauropodomorpha, including basal representatives of this clade (i.e., ‘prosauropods’) as well as basal sauropods and eusauropods. The data matrix included 50 taxa scored across 277 characters (see Appendix S1 and Appendix S2). The initial heuristic tree search resulted in 2360 most parsimonious trees of 619 steps (CI = 0.519, RI = 0.795), found in 939 out of the 1000 replicates. TBR branch swapping of these 2360 trees resulted in a total of 7452 most parsimonious trees (MPTs) of the same tree length.

**Figure 14:**
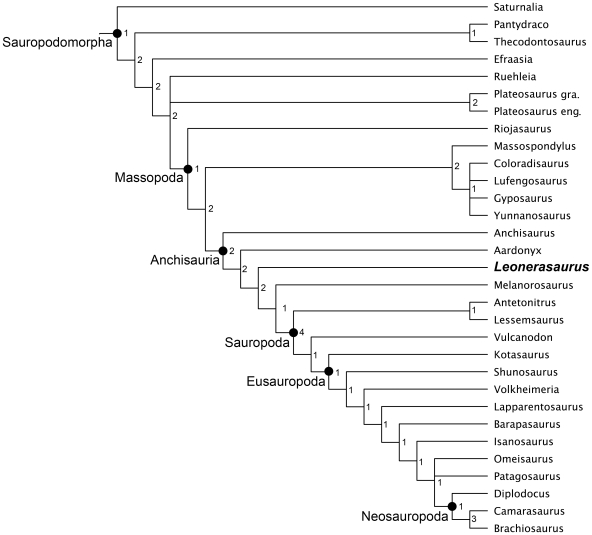
Reduced strict consensus of the phylogenetic analysis. Four unstable taxa (*Jingshanosaurus*, *Blikanasaurus*, *Camelotia*, and *Ferganasaurus*) were excluded from the consensus a posteriori of the heuristic tree searches. Only sauropodomorph taxa are shown (for a complete consensus tree including all outgroup taxa see Appendix S1). Numbers at the nodes represent Bremer support values.

The strict consensus tree has a large polytomy involving basal sauropods and anchisaurian sauropodomorphs (see Appendix S1). However, this is only due to the highly unstable behavior of *Jingshanosaurus xinwaensis* and the fragmentary taxa *Camelotia borealis* and *Blikanasaurus cromptoni*. The instability of the former taxon is due to a mixture of missing data and character conflict, whereas the instability of *Camelotia* and *Blikanasaurus* are exclusively caused by the lack of information (i.e., missing data) and not by character conflict [26], [61]. A reduced consensus tree (see Methods) shows a high degree of resolution along the ‘prosauropod’-sauropod transition, and therefore it is used here to summarize the results of the analysis (Figure 14).

This topology depicts ‘prosauropods’ as a paraphyletic assemblage with respect to Sauropoda, as in most recent phylogenetic analyses of basal sauropodomorphs [12]–[14], [17], [50]. The paraphyly of Prosauropoda and the Late Triassic age of many taxa along the ‘prosauropod’-sauropod transition implies the existence of a large radiation of basal sauropodomorph lineages during the Late Triassic (occurring at least by the Norian). *Leonerasaurus* is interpreted to be an Early Jurassic survivor of this radiation, resembling the case of many other basal sauropodomorphs of Early Jurassic age recorded in other continents (e.g., *Massospondylus, Anchisaurus, Lufengosaurus, Yunnanosaurus,* and *Jingshanosaurus*).

All MPTs depict *Leonerasaurus* as the sister taxon of the clade composed of *Melanorosaurus readi* and Sauropoda (Figure 14). This position is supported by three synapomorphies: sacrum incorporating a caudosacral element (character 150.1), lingual surface of crowns mesiodistally concave (character 104.1), and a low deltopectoral crest in the humerus (character 174.1). The last feature is an unambiguous synapomorphy only in some of the MPTs, given the absence of information in outgroups of this node (e.g., *Aardonyx*).

*Leonerasaurus* is positioned outside the clade of *Melanorosaurus*+Sauropoda (Figure 14), a group recently interpreted as the obligatory “quadrupedal clade” because of the modifications of their hindlimbs and forelimbs [14]. Some of these features are unknown in *Leonerasaurus* and therefore are currently optimized as ambiguous synapomorphies of this clade (e.g., large humerus/femur ratio, presence of deep radial fossa in the ulna). This clade is, however, diagnosed by three unambiguous synapomorphic characters (of which *Leonerasaurus* has the plesiomorphic condition): absence of ventral keels on cranial cervical centra (character 129.1), dorsoventrally deep hyposphenes in dorsal vertebrae (character 145.1), and broad scapular shaft (character 166.1). Additionally, in some of the MPTs, the *Melanorosaurus*+Sauropoda clade is also diagnosed by three other unambiguous synapomorphies of the appendicular skeleton that are absent in *Leonerasaurus*: humeral distal width less than 33% of the humeral length (175.0), absence of well-defined semicircular fossa on the distal flexor surface of the humerus (character 176.1), and cross section of the femoral shaft moderately elongated transversely (character 238.1).

*Leonerasarus* and the recently described transitional sauropodomorph *Aardonyx* are depicted as closer to Sauropoda than *Anchisaurus* (Figure 14), given the presence of six synapomorphic features (see Appendix S1), although only two of them are currently known for *Leonerasaurus*: caudal end of epipophysis in anterior cervicals lacking a free pointed tip (character 122.1) and, pedicles of dorsal neural arches anteroposteriorly long (character 133.1).

#### Phylogenetic robustness and the affinities of *Leonerasaurus*

The phylogenetic position of *Leonerasaurus* has important implications for understanding the origin of Sauropoda (see below). Therefore a thorough evaluation of the robustness of its phylogenetic affinities among Sauropodomorpha is needed to assess the robustness of the inferences made on the MPTs.

Support values are low for most nodes in the reduced consensus (Figure 14), even ignoring the alternative positions of the unstable taxa *Jingshanosaurus*, *Camelotia*, and *Blikanasaurus*. As shown, most nodes of basal sauropodomorphs have Bremer support values of 1 or 2, and only a few nodes have frequency values above 50% in the bootstrap and jackknife analyses (see Appendix S1).

Despite the general low support values, the phylogenetic placement of *Leonerasaurus* is robustly supported within the basal nodes of Anchisauria. Trees depicting *Leonerasaurus* as a non-anchisaurian sauropodomorph require at least 6 extra steps (Templeton p-value = 0.0578), and trees placing the new taxon within Sauropoda require at least 13 extra steps (Templeton p-value = 0.0008). Therefore, the available data strongly indicate that *Leonerasaurus* can be interpreted as a non-sauropod anchisaurian. Furthermore, placing *Leonerasaurus* in a slightly more derived position than in the MPTs (i.e., within the basal nodes of the *Melanorosaurus*+Sauropoda clade) implies between three and six additional steps (depending on the position of other taxa such as *Blikanasaurus* and *Camelotia*; Templeton p-values ranging between 0.0339 and 0.1025). Therefore, the support for placing *Leonerasaurus* as more basal than *Melanorosaurus* is also moderately high.

However, when *Leonerasaurus* is placed more basally within Anchisauria the resultant topologies are only moderately suboptimal, implying two extra steps when it is depicted as more basal than *Aardonyx* or *Anchisaurus*, or as the sister group of either of these taxa (Templeton p-values ranging between 0.1573 and 0.4531). Among these alternative (suboptimal) positions, the possible sister group relationship between *Leonerasaurus* and *Anchisaurus* deserves special attention. The new taxon has two derived features that were up to now unique to *Anchisaurus*: short anteroposterior extension of the medial region of the transverse process of the dorsosacral vertebra (character 154.1 [62]) and preacetabular process of the ilium longer than twice its depth (character 209.1 [59]). Although we must endorse the current most parsimonious placement of *Leonerasaurus* obtained here, future studies and further remains of these taxa are needed to test more thoroughly the putative affinities of *Anchisaurus* and *Leonerasaurus*.

### Body size and ontogenetic stage of MPEF-PV 1663

The ontogenetic stage of the holotype specimen of *Leonerasaurus taquetrensis* is of particular interest due to its small size and its phylogenetic position as a close relative of the large bodied sauropods. Several osteological and histological features suggest that MPEF-PV 1663 is not a juvenile but instead a subadult specimen and that *Leonerasaurus* was, like other basal sauropodomorphs, much smaller than basal sauropods.

#### Ontogenetic stage

Among the osteological features, the most noticeable feature is the complete closure of the suture between the centra and neural arches in most presacral vertebrae. All of the complete cervical vertebrae (axis, c3-c5) have a completely closed neurocentral suture. The posterior cervicals are broken and the neural arches are broken above the level of the neurocentral suture, so it is not possible to assess if these elements had a completely closed neurocentral suture. However, the anterior dorsals have, as the anterior cervicals, completely closed neurocentral sutures. Only a fragment of a posterior dorsal vertebra has a visible trace of the neurocentral suture, but the centrum of this element is nonetheless tightly sutured to the neural arch along an interdigitated suture. The sacral vertebrae also have completely closed neurocentral sutures. Although some regions are broken, there are no evident sutural marks between the transverse processes and the sacral ribs. Finally, the two central sacral elements are fused to each other through their articular surfaces. These osteological features suggest that MPEF-PV 1663 is not a juvenile specimen, although the presence of a visible suture in the posterior dorsal suggests that it may not have reached full skeletal maturity [63], [64]. The lack of fusion of the dorsosacral and caudosacral to the central elements of the sacrum have been interpreted as a sign of skeletal immaturity in sauropods [18], but the caudosacral or dorsosacral elements remain unfused to the primordial sacrals in adult specimens of most basal sauropodomorphs (e.g., *Plateosaurus*, *Massospondylus*, *Lufengosaurus*).

**Figure 15:**
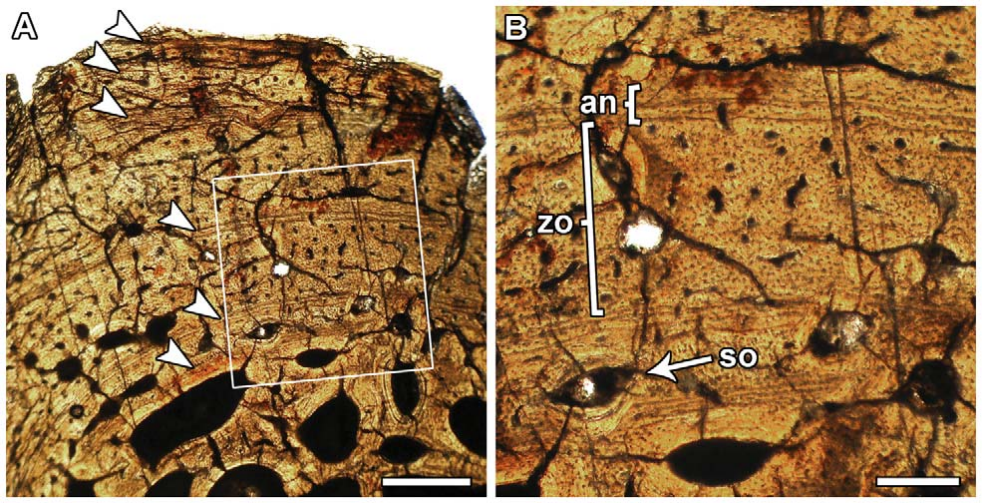
Histological section from a dorsal rib of *Leonerasaurus taquetrensis* (MPEF-PV 1663). A, cortical bone composed of fibrolamellar bone tissue with distinct zones and annuli (arrows). Secondarily enlarged erosion cavities are visible in the perimedullar region. B, Close up of the cortex as indicated in white rectangle in (A) showing the fine structure of zones and annuli. Some secondary osteons are scattered in the inner cortex. Scale bars represent 0.5 (A) and 0.2 mm (B). *Abbreviations: an*, annulus; *so*, secondary osteon; *zo*, zone.

Histological information was obtained from thin sections of the shaft of the femur and a dorsal rib of MPEF-PV 1663 (Figure 15). The transverse sections of the femur show a narrow cortex surrounding a very large medullary cavity. The femur of MPEF-PV 1663 unfortunately suffered poor histological preservation through intensive diagenesis. Although the sample is badly preserved, a distinct cortex composed of primary fibrolamellar bone tissue can be discerned. The fibrolamellar bone is generally highly vascularized and dominated by laminar or irregularly arranged vascular canals. This pattern is observed through the whole cortex, including the outermost cortex. None of the studied sections show evidence of an external fundamental system (EFS), which is a histological proxy for skeletal maturity [65]. The transverse sections of the ribs are better preserved and display a thick cortex surrounding an almost hollow medullary cavity. The perimedullary region exhibits large resorption cavities lined with endosteal lamellar bone tissue. The cortical bone is zonal and contains wide zones separated by annuli and lines of arrested growth (LAGs). The wide zones are composed of primary fibrolamellar bone with mainly longitudinally oriented vascular canals. Six annuli span from the perimedullary region to the outermost cortex. Since annuli and LAGs are assumed to correspond to annual cycles [66], the bone histology of the holotype of *Leonerasaurus taquetrensis* suggest that this individual was at least six years old. This is necessarily a minimum age estimate, because medullary expansion would have removed any additional growth marks originally deposited internal to those observed. The histological analysis thus suggests that MPEF-PV 1663 specimen is neither a young juvenile nor a fully-grown adult.

#### Body size

The preserved axial elements of MPEF-PV 1663 allow an estimate of a total body length of approximately 2.5 meters for this specimen (assuming the presence of 10 cervicals and 15 dorsals as in other basal sauropodomorphs [67], and a caudal region as long as the presacral series). This estimate lies in the lower third of the body length range for basal sauropodomorphs, similar to *Anchisaurus*, *Yunnanosaurus*, and *Coloradisaurus*, but smaller than other basal sauropodomorphs (e.g., *Riojasaurus, Plateosaurus, Lufengosaurus*). Even accepting that fully grown specimens of *Leonerasaurus* may have reached twice the size of the subadult type specimen, such a body length would still be approximately half the length of basal sauropods (e.g., *Lessemsaurus*, *Antetonitrus*, *Vulcanodon*; with estimated body lengths around or above 10 meters long). Furthermore, the body length difference between *Leonerasaurus* and basal sauropods could be even larger, given that the basalmost sauropods are also known from subadult individuals (i.e., all presacral vertebrae of *Lessemsaurus* [11] and *Antetonitrus* [13] specimens have open neurocentral sutures).

**Figure 16:**
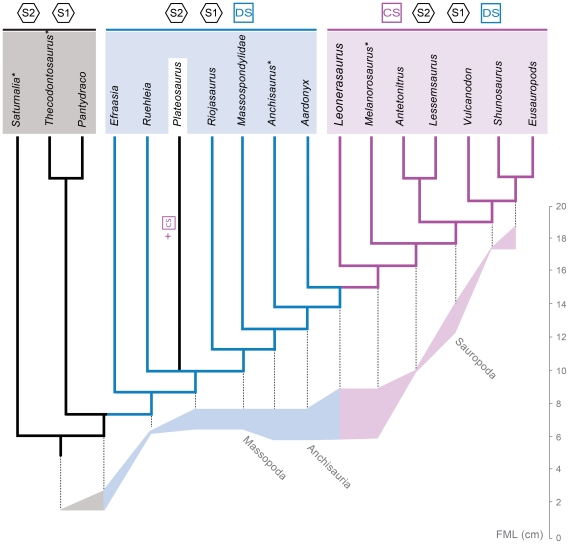
Evolutionary history of the acquisition of sacral vertebrae (above) and body size (below) in basal Sauropodomorpha. Colored boxes and lines on the terminal taxa and branches represent the optimization of the type of sacrum among basal sauropodomorphs. Autapomorphic additions of sacral elements are marked with asterisks (see text for explanation). The curves plotted below the cladogram represent the range of estimated body size (y-axis) in sauropodomorph nodes leading to eusauropods (x-axis). Ancestral reconstructions of body mass are based on femoral lateromedial width (FML; see [69] and Appendix S1 for further data and methods). The terminal ‘Eusauropods’ represents forms more derived than the basal eusauropod *Shunosaurus*, some of which have further increased the sacral count [43], [60].

Other estimates of body size, such as the mediolateral width of the femoral shaft (FML), which has a linear correlation with body mass [68], also indicate that *Leonerasaurus* was a small bodied taxon in comparison with basal sauropods. The FML of MPEF-PV 1663 (4 cm) is similar or smaller than those of other basal sauropodomorphs (e.g., *Anchisaurus, Yunannosaurus, Coloradisaurus*) but is approximately 50% the FML of *Melanorosaurus* and 30% the FML basal sauropods (see Figure 16 and Appendix S1).

In sum, body size estimates for MPEF-PV 1663 place this specimen in the lower third of the body size variation of basal sauropodomorphs. As the available osteological and histological data suggest that MPEF-PV 1663 is a subadult specimen that has not reached cessation of growth, it is likely that *Leonerasaurus* did not differ markedly in body size from other basal sauropodomorphs. This is consistent with the body size optimized at the ancestral node of *Leonerasaurus* (see below and Figure 16). Therefore, despite the subadult condition of the holotype we postulate that the body size difference between *Leonerasaurus* and basal sauropods was at least as large as those noted between other basal sauropodomorphs and sauropods ([68], [69]; see below).

### Evolutionary Origins of the Sauropod-type Sacrum

The increase of sacral vertebrae is a common evolutionary trend present in the three major groups of Dinosauria (Ornithischia, Theropoda, Sauropodomorpha) [70]. Within Sauropodomorpha, the number of sacral vertebrae has long been recognized to increase along the evolutionary history of the group, and one of the features that traditionally diagnosed the large-bodied Sauropoda was the presence of four sacral vertebrae (see [43], [69], [71]).

However, the precise pattern of sacral evolution in the early evolution of Sauropodomorpha has been debated in recent years. Most of this debate has been focused on the homology of different sacral vertebrae in basal sauropodomorphs, discussed at length in recent contributions [16], [18], [56], [57], [72]–[77]. Although some disagreements still exist over the homology of the sacral vertebrae of some taxa (e.g., *Anchisaurus*; see below), most of the authors mentioned above currently agree in the homology of the sacral elements of basal sauropodomorphs.

Despite the uncertainties and low support values noted above for the phylogenetic results, the most parsimonious hypotheses retrieved in this analysis recognize three major stages (Figure 16) in the early evolutionary history of the sauropodomorph sacrum: 1) the plesiomorphic condition for Sauropodomorpha in which the sacrum is almost exclusively composed of the two primordial sacrals (S1+S2); 2) the condition present in most basal sauropodomorphs (‘prosauropods’) characterized by the incorporation of a third element identified as a dorsosacral (DS+S1+S2); 3) the condition of the large-bodied Sauropoda that is characterized by the presence of four sacrals (or more in derived taxa), in which one caudosacral element is incorporated (DS+S1+S2+CS) [18], [50].

Recently described specimens of the near-sauropod *Melanorosaurus readi* [12], [76] have shown that the four-sacral condition is not diagnostic of Sauropoda, but instead of the more inclusive clade of *Melanorosaurus*+Sauropoda [14]. In fact, the presence of four sacrals (as well as other characters such as the development of an eccentric femoral shaft) has been regarded as possible a adaptation to support increasing gut volumes and body masses in this clade of large-bodied sauropodomorphs [14].

The presence of four sacrals (DS+S1+S2+CS) in *Leonerasaurus*, coupled with its smaller body size and its position as the sister group of the large bodied clade of *Melanorosaurus*+Sauropoda, is highly significant for understanding the origin of the sauropod-type sacrum. The optimization of sacral configuration along the evolutionary history of basal Sauropodomorpha (Figure 16) shows that the four sacrals that previously diagnosed Sauropoda (or the clade of large bodied taxa composed by *Melanorosaurus*+Sauropoda) actually appeared earlier in the evolutionary history of the group, being diagnostic of a more inclusive clade. Furthermore, the four-sacral condition of *Leonerasaurus* not only reveals an earlier phylogenetic origin of this feature but also shows that the appearance of the sauropod-type of sacrum predated the body size increase that characterizes the origin of Sauropoda (Figure 16).

Thus, the new information provided by *Leonerasaurus* (and other recently described forms) allows a more complete understanding of the evolutionary history of Sauropodomorpha. The early evolution of this group (and the origins of Sauropoda) is certainly characterized by a trend of increasing both body size and sacral count. However, the decoupled evolution of these two features seems to go against an adaptive argument to explain the appearance of an increase in the number of sacral vertebrae as a response to the body size increase in Sauropoda (and closely related forms).

Considering the alternative (suboptimal) phylogenetic positions for *Leonerasaurus* within Anchisauria (see above), the presence of four sacrals in this species would also be significant. If *Leonerasaurus* is indeed a more basal anchisaurian sauropodomorph (e.g., the sister group of *Anchisaurus*), its sacral configuration must be explained as an evolutionary convergence between the new taxon and sauropods (paralleling the addition of a fourth sacral element). Such a convergent pattern would be unique among basal sauropodomorphs and would also indicate an increase in sacral number decoupled from an increase in body size along the lineage of *Leonerasaurus*.

#### Homoplasy and Conflictive Homology

The gradual acquisition of sacral vertebrae along the evolutionary history of Sauropodomorpha is, however, not free of homoplasy. Several instances of convergences and reversals need to be postulated in order to explain the sacral configuration of some basal sauropodomorphs in the most parsimonious trees (as well as in any of the previously published phylogenies of basal sauropodomorphs; see below).

The clearest case is present in *Plateosaurus*, which has a unique sacral configuration among basal sauropodomorphs [56], [57], composed by the two primordial sacrals plus a caudosacral (S1+S2+CS; Figure 16). Given that *Plateosaurus* is nested within sauropodomorphs with a DS+S1+S2 sacral configuration, the incorporation of a caudosacral and the deletion of a dorsosacral must be interpreted as autapomorphic transformations of the *Plateosaurus* lineage (an event explained as a possible homeotic frame-shift [75]).

Three other cases of possible homoplastic transformations can be mentioned, although the extent of these homoplasies actually depends on the debated interpretation of some incomplete or poorly preserved sacra.

First, the three sacrals of *Anchisaurus* have been interpreted either as DS+S1+S2 [12], [59] or as S1+S2+CS [16], [50]. The former interpretation is congruent with the position of *Anchisaurus* in the analysis presented here (as well as in other phylogenetic studies [12], [50], [59]), given that this taxon is bracketed by forms with a DS+S1+S2 configuration. The second interpretation, however, would imply that (as in the case of *Plateosaurus*) a caudosacral element was incorporated and a dorsosacral was eliminated from the sacrum along the terminal branch leading to *Anchisaurus*. As noted above, if *Leonerasaurus* is indeed closer to *Anchisaurus* than to sauropods, even more sacral modifications would characterize this clade of small sauropodomorphs.

Second, one of the specimens referred to the near-sauropod *Melanorosaurus readi* (NM QR1551) has preserved four sacral vertebrae that were originally interpreted as DS+S2+S1+CS [76], but have been recently reinterpreted as two dorsosacrals followed by the two primordial sacrals [12] (A. Yates, pers. com.). Another specimen referred to *Melanorosaurus readi* (NM QR3314) has preserved, instead, five sacral elements. Comparisons between the two specimens suggest that the complete sacral configuration of *Melanosaurus readi* includes two dorsosacrals, followed by the two primordial sacrals, and a single caudosacral (A. Yates, pers. com.). This condition must be interpreted as autapomorphic, given the presence of four sacrals in basal sauropods (and *Leonerasaurus*). The disparity among referred specimens of *Melanorosaurus readi* is likely due to the incompleteness of the specimen NM QR1551, but further studies on these specimens are needed to clarify this problem.

Third, the basalmost sauropodomorph taxa *Saturnalia* and *Thecodontosaurus* clearly have two major sacral elements that are extensively attached to the iliac blade. However, a marginal participation of a caudosacral may be present in these forms [55], [78]. The lateral contact of this putative accessory caudosacral has not been preserved in any of the specimens, and these inferences are mostly based on rugose surfaces that might represent areas of attachment of a caudosacral element. The presence of a caudosacral in these basal taxa needs to be confirmed with more complete remains.

The evolution of the sacrum in basal sauropodomorphs, therefore, shows a complex pattern of character evolution, and the long recognized trend of increase in sacral count might have occurred convergently in several lineages of Sauropodomorpha since their earliest evolutionary history. Such a complex pattern of sacral evolution (e.g., acquisition of CS in *Plateosaurus* or an autapomorphic fifth sacral in *Melanorosaurus*) is not only implied by the results of the present phylogenetic analysis. These instances of homoplasy are similarly implied by the topologies of all phylogenetic analyses of basal sauropodomorphs published in recent years [12]–[14], [16]–[17], [19], [46], [48], [50], [59].

Irrespective of these parallel trends and the phylogenetic uncertainties, our results show that the trunk lineage leading to Sauropoda seems to have gradually increased the number of sacral vertebrae, with sauropods inheriting a sacrum with four vertebrae from their more primitive relatives. The large number of recent discoveries and phylogenetic studies of basal sauropodomorphs offer a wealth of new information about their early evolution, providing critical evidence to understand the pattern and processes acting in one of the major evolutionary transformations of Dinosauria, the origins of Sauropoda. In this sense, the findings reported here support a gradual acquisition of sacral characters along the phylogenetic line leading to Sauropoda. This pattern is paralleled by recent studies that showed gradual acquisition of characters in the forelimb of this lineage [10], suggesting that many of the numerous features that previously distinguished Sauropoda from other dinosaurs appeared gradually in the evolutionary history of Sauropodomorpha.

## Supporting Information

Appendix S1 Supplementary phylogenetic information, including character list, data matrix, strict consensus trees, complete list of synapomorphies of the nodes present in the strict consensus and reduced strict consensus, and further data on support measures. Additionally, supplementary information, data, and methods relevant to Figure 16 are provided.
(0.88 MB DOC)

Appendix S2 Nexus file of the data matrix used in the phylogenetic study.
(0.02 MB TXT)

Table S1 Selected measurements of *Leonerasaurus taquetrensis* (MPEF-PV 1663).
(0.07 MB DOC)
