# Supplementary material for: A New Sauropodomorph Dinosaur from the Early Jurassic of Patagonia and the Origin and Evolution of the Sauropod-type Sacrum
Source: PLoS One. 2011 Jan 26;6(1):e14572. doi: 10.1371/journal.pone.0014572 (PMC3027623; doi:10.1371/journal.pone.0014572)
Supplement: Appendix S1 — Supplementary phylogenetic information, including character list, data matrix, strict consensus trees, complete list of synapomorphies of the nodes present in the strict consensus and reduced strict consensus, and further data on support measures. Additionally, supplementary information, data, and methods relevant to Figure 16 are provided. (0.88 MB DOC) [file pone.0014572.s001.doc]

**Supporting Information – Appendix S1**

**A New Sauropodomorph Dinosaur from the Early Jurassic of Patagonia and the Origin and Evolution of the Sauropod-type Sacrum**

**Diego Pol^1^*, Alberto Garrido^2^**

**1** CONICET, Avenida Fontana 140, Museo Paleontológico Egidio Feruglio, Trelew 9100, Chubut, Argentina, **2** Dirección de Minería de la Provincia de Neuquén, Museo Dr. Olsacher, Echeverri y Ejercito Argentino, Zapala 8340, Neuquén, Argentina

*1. Supplementary phylogenetic information*

*1.a Character list*

*1.b Data matrix*

*1.c List of taxa and specimens examined*

*1.d Strict consensus tree and list of synapomorphies*

*1.e Reduced strict consensus and additional list of synapomorphies*

*1.f Support measures*

*2. Supplementary information to Figure 16*

*2.a Data*

*2.b Methods*

*3. Supplementary references*

**1. Supplementary phylogenetic information**

**1.a Character list.** The complete list of characters used in the phylogenetic analysis is provided here. The following multistate characters were treated as ordered:

16, 18, 46, 56, 65, 97, 113, 117, 118, 125, 127, 132, 133, 135, 136, 138, 143, 147, 148, 165, 170, 174, 187, 196, 198, 210, 242, 253, 257

Character 1. Skull length relative to femoral length (Gauthier, 1986).

0: longer than two thirds of the femoral length

1: shorter than two thirds of the femoral length

Character 2. Lateral plates appressed to the labial side of teeth (Upchurch, 1995)

0: absent

1: present

Character 3. Distal end of dorsal premaxillary process (Sereno, 1999)

0: tapered

1: transversely expanded

Character 4. Anterior margin of premaxilla

0: oriented subvertically, forming an angle of 70 to 90 degrees with

buccal margin

1: oriented posterodorsally, forming an angle of 45 degrees with

buccal margin

Character 5. Profile of premaxilla (Upchurch, 1995)

0: convex

1: with an inflection at the base of the dorsal process

Character 6. Premaxillary main body shape

0: subrectangular, anteroposteriorly elongated

1: subquadrangular

Character 7. Caudolateral process of premaxilla (Yates and Kitching, 2003)

0: present

1: absent

Character 8. Ratio of narial diameter to orbital diameter (Wilson and Sereno, 1998)

0: less than 0.5

1: greater than 0.5

Character 9. Narial position (Wilson and Sereno, 1998)

0: near terminus of snout

1: retracted caudodorsally

Character 10. Perinarial fossa. (Sereno, 1999; Langer, 2004)

0: absent or shallow

1: expanded in the anteroventral corner of naris

Character 11. Anteroventral margin of perinarial fossa (Yates, 2007)

0: well developed sharp ridges

1: poorly defined edges

Character 12. Subnarial gap (Gauthier, 1986)

0: absent

1: present

Character 13. Subnarial foramen (Sereno, 1999)

0: absent

1: present

Character 14. Location of subnarial foramen (Yates, 2007)

0: small with the internal opening just above the tooth row

1: large, opening into the palata well above the tooth row

Character 15. Internarial bar (Galton and Upchurch, 2004)

0: wide (transverse width equals or exceeds rostrocaudal width)

1: laterally compressed (transverse width is less than rostrocaudal

width)

Character 16. Contact between caudolateral process of premaxilla and rostroventral process of nasal (Gauthier, 1986)

0: broad sutured contact

1: point contact

2: separated by maxilla

Character 17. Orientation of maxillary ascending process

0: Posteriorly deflected along its dorsal half

1: Posteriorly deflected along its dorsalmost fifth

Character 18. Rostral margin of the maxilla and its ascending process (Langer, 2004)

0: straight, or convex

1: concave, with the base of the ascending process continuous with

the rostral margin of the bone

2: markedly offset from it

Character 19. Length of rostral ramus of the maxilla (Sereno, 1999)

0: less than its dorsoventral depth

1: greater than its dorsoventral depth

Character 20. Anterior profile of maxilla (Upchurch, 1995)

0: slopes continuously towards the rostral tip

1: with strong inflection

Character 21. Size of the neurovascular foramen at the caudal end of the lateral maxillary row (Yates, 2003)

0: not larger than others

1: distinctly larger than others

Character 22. Direction of the neurovascular foramen at the caudal end of the lateral maxillary row (Sereno, 1999)

0: rostrally, ventrally, laterally

1: caudally

Character 23. Arrangement of lateral maxillary neurovascular foramina (Sereno, 1999)

0: linear

1: irregular

Character 24. Shape of the rostral margin ofthe antorbital fenestra (Yates and Kitching, 2003)

0: strongly concave, creating a narrow antorbital fossa

1: straight to gently concave, creating a broad subtriangular antorbital

fossa

Character 25. Medial wall of the antorbital fossa extends (Sereno, 1999)

0: through the entire ventral border of the antorbital fenestra

1: does not reach the caudoventral corner of the fenestra

Character 26. Ventral rim of the antorbital fossa (Langer, 2004)

0: parallel to tooth row

1: ventrally sloped in its caudal part

Character 27. Rostrocaudal length of the antorbital fossa (Yates, 2003)

0: greater than that of the orbit

1: less than that of the orbit

Character 28. Dorsally open neurovascular canal on the floor of the antorbital fossa (Yates and Kitching, 2003)

0: absent

1: present

Character 29. Nasal contribution to the margin of the antorbital fenestra (Sereno, 1999)

0: absent

1: present

Character 30. Dorsal profile of snout (Yates, 2007)

0: straight or gently convex

1: with a depression behind the naris

Character 31. Caudal margin of the conjoined nasals in dorsal view (Yates, 2003)

0: concave or W-shaped

1: convex

Character 32. Ventral ramus of the lacrimal (Langer, 2004)

0: short and mainly inclined

1: long, forming about 0.75 or more of the maximum preorbital skull

height

Character 33. Descending process of lacrimal

0: curved, subvertically oriented (at its dorsal half)

1: straight and obliquely oriented along its entrire length

Character 34. Length of the rostral ramus of the lacrimal (Yates, 2007)

0: greater than half the length of the ventral ramus

1: less than half the length of the ventral ramus

Character 35. Dorsal exposure of the lacrimal (Gauthier, 1986)

0: present

1: absent

Character 36. Dorsoventral extension of lacrimal antorbital fossa (Langer, 2004)

0: through more than half of the bone height

1: is restricted to the ventral half of the bone

Character 37. Extension of the antorbital fossa onto the ventral end of the lacrimal (Yates and Kitching, 2003)

0: present

1: absent

Character 38. Lacrimal at caudal/dorsocaudal part of antorbital fenestra (Sereno, 1999; Langer, 2004)

0: does not fold

1: folds over the caudal/dorsocaudal part of antorbital fenestra

Character 39. Jugal contribution to the antorbital fenestra (Galton and Upchurch, 2004)

0: absent

1: present

Character 40. Rostral ramus of jugal

0: expands rostrally, forming part of the rostral margin of the orbit

1: does not expand rostrally

Character 41. Shape of the rostral end of the jugal (Yates and Kitching, 2003)

0: blunt

1: sharply tapered

Character 42. Extension of postorbital posterior branch

0: extensive, reaching the posterodorsal corner of infratemporal

fenestra

1: reduced, not reaching the posterodorsal corner of infratemporal

fenestra

Character 43. Curvature of postorbital posterior branch

0: straight

1: medially bowed, laterally concave in dosal view

Character 44. Transverse width of the ventral ramus of the postorbital (Wilson and Sereno, 1998)

0: less than its rostrocaudal width at mid shaft

1: greater than its rostrocaudal width at mid shaft

Character 45. Ventral end of descending process of postorbital

0: straight, ventrally direceted tip

1: anteriorly deflected ventral tip

Character 46. Position of the rostral margin of the lower temporal fenestra (Upchurch, 1995)

0: behind the orbits

1: extends under the rear half of the orbit

2: extends as far forward as the midlength of the orbit

Character 47. Prefrontal posteromedial extension on the dorsal surface of the skull

0: small

1: enlarged

Character 48. Length of the caudal process of the prefrontal (Yates and Kitching, 2003)

0: short

1: elongated

Character 49. Frontal contribution to supratemporal fossa (Gauthier, 1986)

0: present

1: absent (excluded by a parietal-postorbital contact)

Character 50. Frontal-parietal suture

0: transversely oriented

1: V-shaped, posteriorly directed

Character 51. Orientation of the long axis of the supratemporal fenestra (Wilson and Sereno, 1998)

0: longitudinal

1: transverse

Character 52. Length of the quadratojugal ramus of the squamosal relative to the width at its base (Sereno, 1999)

0: less than four times its width

1: greater than four times its width

Character 53. Ventral ramus of squamosal forms (Langer, 2004)

0: more than half of the caudal border of the lower temporal fenestra

1: or less than half of the caudal border of the lower temporal fenestra

Character 54. Squamosal-quadratojugal contact (Gauthier, 1986)

0: present

1: absent

Character 55. Angle between the rostral and dorsal rami of the quadratojugal (Galton and Upchurch, 2004)

0: is 90> or more

1: is approximately 60> or less

Character 56. Dorsal ramus of the quadratojugal

0: longer than the rostral ramus

1: the same length than the rostral ramus

2: shorter than the rostral ramus

Character 57. Shape of the rostral end of the jugal ramus of the quadratojugal (Wilson and Sereno, 1998)

0: tapered

1: dorsoventrally expanded

Character 58. Proportion of the height of the quadrate that is occupied by the pterygoid wing (Yates and Kitching, 2003)

0: at least 70%

1: less than 70%

Character 59. Ectopterygoid ventral recess (Gauthier, 1986)

0: absent

1: present

Character 60. Shape of jugal process of ectopterygoid (Yates and Kitching, 2003)

0: gently curved

1: strongly recurved and hook-like

Character 61. Ectopterygoid-jugal contact

0: present

1: absent

Character 62. Palatine-ectopterygoid suture on palate

0: absent, separated by pterygoid

1: present

Character 63. Palatine shaft

0: extensive, being more than half the length of the medial expansion

1: reduced, being less than 40% of the length of the medial expansion

Character 64. Position of the maxillary articulating surface of the palatine (Yates and Kitching, 2003)

0: along the lateral margin of the bone

1: at the end of a narrow anterolateral process

Character 65. Medial process of the pterygoid forming a hook around the basipterygoid process (Yates and Kitching, 2003)

0: absent

1: flat and blunt-ended

2: bent upwards and pointed

Character 66. Ridge formed along the junction of the parabasisphenoid and the basioccipital, between the basal tuberae (Yates and Kitching, 2003)

0: present with a smooth rostral face

1: present with a median fossa on the rostral face

2: absent with the basal tuberae being separated by a deep caudally

opening U-shaped fossa

Character 67. Ossification of the extremity of the basal tuber (Yates and Kitching, 2003)

0: complete so that the basioccipital and parabasispenoid form a

single rugose tuber

1: unossified with the basioccipital forming a ventrally facing platform

of unfinished bone that abuts a similarly unfinished, caudally facing

wall of the parabasisphenoid

Character 68. Shape of basal tuberae (Yates and Kitching, 2003)

0: knob-like, with basisphenoidal component rostral to basioccipital

component

1: forming a transverse ridge with the basisphenoidal component

lateral to the basioccipital component

Character 69. Dorsoventral depth of the parasphenoid rostrum (Yates and Kitching, 2003)

0: much less than its transverse width

1: about equal to its transverse width

Character 70. Shape of the floor of the braincase in lateral view (Galton and Upchurch, 2004)

0: relatively straight with the basal tubera, basipterygoid processes and parasphenoid rostrum roughly aligned

1: bent with the basipterygoid processes and the parasphenoid rostrum below the level of the basioccipital condyle and the basal tuberae

2: bent with the basal tuberae lowered below the level of the basioccipital and the parasphenoid rostrum raised above it

Character 71. Deep transverse wall of bone between basipterygoid processes (Galton and Upchurch, 2004)

0: absent

1: present

Character 72. Length of the basipterygoid processes (Benton et al., 2000)

0: less than the height of the braincase

1: greater than the height of the braincase

Character 73. Location of the posttemporal fenestra (Yates and Kitching, 2003)

0: between the parietal, the supraoccipital and the exoccipital-opisthotic complex

1: fully enclosed by the suproccipital

Character 74. Fontanelle between the supraoccipital and the parietals (Yates and Kitching, 2003)

0: absent

1: present

Character 75. Paroccipital process, in caudal view, projects (Rauhut, 2003)

0: laterally or dorsolaterally

1: ventrolaterally

Character 76. Shape of the supraoccipital (Yates, 2003)

0: diamond-shaped, (at least as high as wide)

1: semilunate (and wider than high)

Character 77. Shape of upper jaws in ventral view (Wilson and Sereno, 1998)

0: narrow with an acute rostral apex

1: broad and U-shaped

Character 78. Dorsoventral expansion at the symphyseal end of the dentary (Wilson and Sereno, 1998)

0: absent

1: present

Character 79. Dentary symphysis (Sereno, 1999)

0: restricted to the rostral margin of the dentary

1: expanded along the ventral border of the bone

Character 80. Anterior end of dentary in lateral view (Galton and Upchurch, 2004)

0: substraight or curves slightly upwards towards its rostral tip

1: dorsal margin curves ventrally towards its rostral tip

2: ventral and dorsal margins curve ventrally

Character 81. Height/length ratio of the dentary (Benton et al., 2000)

0: less than 0.2

1: greater than 0.2

Character 82. Ridge on lateral surface of the dentary (possibly associated with a fleshy cheek in life) (Gauthier, 1986; Galton and Upchurch, 2004)

0: absent

1: present

Character 83. Splenial mylohyoid foramen (Rauhut, 2003)

0: absent

1: present

Character 84. Intramandibular joint (Sereno and Novas, 1992)

0: absent

1: present

Character 85. Retroarticular process length (Galton and Upchurch, 2004)

0: short

1: long

Character 86. Retroarticular process in lateral and dorsal view (Yates, 2003)

0: does not taper caudally

1: tapers caudally

Character 87. Strong medial embayment behind glenoid of the articular in dorsal view (Yates and Kitching, 2003)

0: absent

1: present

Character 88. Craniomandibular joint position (Sereno, 1999)

0: at about the same level of the tooth rows

1: set well bellow the tooth rows

Character 89. Orientation of maxillary (or posterior) tooth crowns (Gauthier, 1986)

0: erect

1: procumbent

Character 90. Orientation of dentary tooth crowns (Gauthier, 1986)

0: erect

1: procumbent

Character 91. Number of teeth in the premaxilla (Galton and Upchurch, 2004)

0: four

1: five or more

Character 92. Number of dentary teeth (Wilson and Sereno, 1998)

0: 17 or fewer

1: 18 or more

Character 93. Adjacent tooth crowns (Galton and Upchurch, 2004)

0: are aligned so they do not overlap in lateral view

1: are angled relative to the long-axis of the jaw so tooth crowns appear to overlap in lateral view (each tooth has its mesial margin lying lingual to the distal margin of the crown immediately in front)

Character 94. First dentary tooth (Sereno, 1999)

0: lies at the extreme rostral end of the dentary

1: is inset a short distance from the rostral tip of the dentary

Character 95. Tooth crowns on the rostral quarter of the tooth bearing areas of the upper and lower jaws (Gauthier, 1986)

0: about the same height than more caudal teeth

1: significantly higher than more caudal teeth

Character 96. Maxillary/dentary tooth crowns curvature (Sereno, 1999)

0: caudally curved

1: not curved

Character 97. Lanceolate tooth crowns (Sereno, 1999)

0: absent

1: present together with some non-lanceolate crowns

2: encompasses all dental elements of maxilla and dentary

Character 98. Maximum width of lanceolate tooth crowns

0: maximum width located at the base of the crown, just above the tooth-crown construction

1: maximum width located at the midpoint of crown’s height

Character 99. Basal constriction in teeth (Langer, 2004)

0: absent

1: present in most maxillary/dentary tooth crowns

Character 100. Tooth crown serrations (Benton et al., 2000)

0: project approximately perpendicular to the long-axis of the crown

1: project at approximately 45> to the long-axis of the crown

Character 101. Distribution of the serrations along the mesial and distal carinae of the tooth (Yates and Kitching, 2003)

0: extend along most of the length of the crown

1: restricted to the distal half of the crown

Character 102. Prominent grooves near the mesial and distal margins of the labial surface of each tooth crown (Upchurch, 1998)

0: absent

1: present

Character 103. Labial and lingual surface of maxillary/dentary tooth crowns (Langer, 2004)

0: evenly convex

1: bearing marked low eminence

Character 104. Lingual surfaces of tooth crowns (Upchurch, 1995)

0: are convex or nearly flat mesiodistally

1: are concave mesiodistally

Character 105. Texture of the enamel surface external surface (Wilson and Sereno, 1998)

0: smooth

1: finely wrinkled texture

2: coarsely wrinkled texture

Character 106. Tooth-tooth occlusion (Wilson and Sereno, 1998)

0: absent

1: present

Character 107. Tooth implantation

0: roots unfused to alveolar walls

1: roots of erupted teeth partially fused to alveolar walls

Character 108. Length of the atlantal intercentrum (Yates and Kitching, 2003)

0: greater than that of the axial intercentrum

1: shorter than that of the axial intercentrum

Character 109. Articular facet for the atlas in axial intercentrum (Gauthier, 1986)

0: saddle-shaped, craniocaudally concave and lateromedially convex

1: concave, with upturned lateral border

Character 110. Axial prezygapophyses and postzygapophyses (Yates and Kitching, 2003)

0: equidistant from the midline of the axis

1: postzygapophyses set wider than the prezygapophyses

Character 111. Short cranially projected pedicels bearing axial prezygapophyses (Yates and Kitching, 2003)

0: absent

1: present

Character 112. Axial postzygapophyses posterior margin (Sereno, 1999)

0: project caudally beyond end of centrum

1: are flush with end of centrum

Character 113. Number of cervical vertebrae (Wilson and Sereno, 1998)

0: nine

1: ten

2: eleven

3: twelve

4: 13 or more

Character 114. Strong lateral compression of the cranial cervical vertebrae (Upchurch, 1998)

0: absent

1: present

Character 115. Neural spine transversely wide on posterior cervicals

0: absent

1: incipient

2: markedly expanded

Character 116. Postzygaodiapophyseal lamina on cervical neural arches 4 to 8 (Yates, 2003)

0: present

1: absent

Character 117. Depression on anterior surface of pedicels of cervical neural arches

0: absent or poorly defined

1: small but well delimited

2: deep

Character 118. Infrapostzygapophyseal constriction on cervicals

0: absent

1: incipient

2: well developed

Character 119. Cervical epipophyses (Novas, 1996)

0: absent

1: present

Character 120. Epipophyses on caudal cervical vertebrae (6-9) (Gauthier, 1986).

0: absent

1: present

Character 121. Epipophyses overhanging the rear margin of the postzygapophyses (Yates, 2003)

0: present in at least some postaxial cervical vertebrae

1: absent

Character 122. Caudal ends of cranial, postaxial epipophyses (Yates and Kitching, 2003)

0: with a free pointed tip

1: joined to the postzygapophysis along their entire length

Character 123. Infradiapophyseal lateral fenestra

0: absent

1: present

Character 124. Pleurocelous in cervical neural arches

0: absent

1: present

Character 125. Lateral depression-pleurocoels on cervical centra (Gauthier, 1986)

0: slightly marked and simple

1: well defined with sharp anterior edge

2: well defined as a pleurocoel

Character 126. Length of centra of postaxial cranial cervical vertebrae (3-5) with respect to the axis (Sereno, 1999)

0: subequal than that of the axis

1: longer than that of the axis

Character 127. Elongation of mid cervical centrum (3-5) (Upchurch, 1998)

0: less than 3.0 times the centrum height

1: 3.0-4.0 times the centrum height

2: >4.0 times the centrum height

Character 128. Centra of caudal cervical vertebrae (6-8) (Gauthier, 1986)

0: subequal than those of cranial trunk vertebrae

1: longer than those of cranial trunk vertebrae

Character 129. Ventral keels on cranial cervical centra (Upchurch, 1998)

0: present

1: absent

Character 130. Articulations between cervical centra (Gauthier, 1986)

0: amphicoelous-amphiplatyan

1: opisthocoelous (i.e. a cranial hemispherical convexity articulates with a corresponding concavity on the caudal surface of the preceding cervical)

Character 131. Mid-cervical ribs (Sereno, 1999)

0: short and directed caudoventrally

1: long and sub-parallel to the neck

Character 132. Neural arch/vertebra height in dorsals (Bonaparte, 1999)

0: 0.5 or less

1: between 0.60 and 0.75

2: 0.8 or more

Character 133. Anteroposterior length of dorsal neural arch pedicels (Bonaparte, 1999)

0: 50% centrum height

1: 75%-80% centrum height

2: =>100% centrum height

Character 134. Dorsal neural spine (Bonaparte, 1999)

0: low

1: high

Character 135. Dorsal neural spine cross section (Bonaparte, 1999)

0: laminar

1: broad, subtriangular

2: cross-shaped in cross section

Character 136. Laterally expanded tables at the midlength of the distal surface of the neural spines (Yates and Kitching, 2003)

0: absent in all vertebrae

1: present on the pectoral vertebrae

2: present on the pectoral and cervical vertebrae

Character 137. Transverse process respect to sagittal plane (Bonaparte, 1999)

0: perpendicular

1: dorsal deflected

Character 138. Depression on dorsolateral surface of diapophyseal lamina (Bonaparte, 1999)

0: absent

1: slightly marked

2: deeply marked

Character 139. Spinodiapophyseal lamina on middle and caudal dorsal vertebrae (Wilson and Sereno, 1998)

0: absent

1: present

Character 140. Diapo-prezygapophyseal lamina and associated anterior triangular fossa (chonos) (Yates and Kitching, 2003)

0: present on all dorsals

1: absent in mid dorsals

Character 141. Excavations of the cranial face of the dorsal neural arches, surrounding the neural canal (Bonaparte, 1999; Yates and Kitching, 2003)

0: absent

1: present

Character 142. Well-developed suprapostzygapophyseal laminae (Bonaparte, 1999)

0: absent

1: present on the caudal dorsal vertebrae

2: present on all dorsal vertebrae

Character 143. Spinoprezygapophyseal laminae (Yates and Kitching, 2003)

0: absent

1: present as low ridges on caudal dorsal vertebrae

2: present as thin laminae on most dorsals

Character 144. Hyposphene-hypantrum articulation in trunk vertebrae (Gauthier, 1986)

0: absent

1: present

Character 145. Dorsoventral height of the hyposphenes (Yates, 2007)

0: much less than the dorsoventral height of the neural canal

1: equal to the dorsoventral height of the neural canal

Character 146. Parapophyses contact with the centrum in vertebrae caudal to the twelfth presacral element (Langer, 2004)

0: do not contact

1: contact

Character 147. Pleurocelous in dorsal centra (Bonaparte, 1999; Wedell, 2003)

0: acamerate, with no pneumatic fossa (or shallow concavities)

1: acamerate with deep sharply delimited pneumatic fossa

2: procamerate

3: camerate

Character 148. Articular centra of dorsal vertebrae (Bonaparte, 1999)

0: amphicoelous or amphiplatyan

1: opisthocoelous up to D2

2: opisthocoelous up to mid-dorsals

3: opisthocoelous up to sacrals

Character 149. Last presacral rib (Yates and Kitching, 2003)

0: free

1: fused to vertebra

Character 150. Caudosacral vertebra (Galton and Upchurch, 2004)

0: absent

1: present

Character 151. Dorsosacral vertebrae (Gauthier, 1986)

0: absent

1: one dorsosacral

2: more than one dorsosacral

Character 152. Strong constriction between the sacral rib and the transverse process of the first primordial sacral rib (and dorso-sacral if present) in dorsal view (Yates and Kitching, 2003)

0: absent

1: present

Character 153. Transverse processes of sacral vertebrae (Langer, 2004)

0: as broad as the ribs of the respective vertebra

1: expanded, roofing the space between adjacent ribs

Character 154. Anteroposterior extension of medial region of transverse process of dorsosacral (Yates, 2010)

0: more than 50% the centrum length

1: less than 50% the centrum length

Character 155. Ribs of the two primordial sacrals (Langer, 2004)

0: cover the entire medial surface of the iliac alae

1: shorter than the iliac alae

Character 156. Sacricostal yoke (distal ends of sacral ribs fuse together) (Wilson and Sereno, 1998)

0: absent

1: present

Character 157. Length of base of the proximal caudal neural spines (Yates and Kitching, 2003)

0: greater than half the length of the neural arch

1: less than half the length of the neural arch

Character 158. Position of postzygapophyses in proximal caudal vertebrae (Yates and Kitching, 2003)

0: protruding with an interpostzygapophyseal notch visible in dorsal view

1: placed on either side of the caudal end of the base of the neural spine without any interpostzygapophyseal notch

Character 159. Caudal "hyposphenal" ridge (Upchurch, 1995)

0: absent

1: present

Character 160. Centrum length divided by centrum height (in the most cranial caudals) (Gauthier, 1986)

0: is approximately 1.0 or more

1: is approximately 0.5-0.6

Character 161. Length of midcaudal centra (Yates and Kitching, 2003)

0: greater than twice the height of their proximal faces

1: less than twice the height of their proximal faces

Character 162. Longitudinal ventral sulcus on caudal centra (Upchurch, 1995)

0: absent

1: present

Character 163. Length of the longest chevron (Yates and Kitching, 2003)

0: less than the length of the preceding centrum

1: greater than the length of the preceding centrum

Character 164. Longitudinal ridge on the dorsal surface of the sternal plate (Yates and Kitching, 2003)

0: absent

1: present

Character 165. Scapular blade expansion (Pol and Powell, 2007)

0: poorly expanded, with its minimal craniocaudal breadth equal or greater than half that of its dorsal margin

1: moderately expanded dorsally

2: remarkably expanded dorsally, as much as the ventral end

Character 166. Minimum width of the scapula (Gauthier, 1986)

0: less than 20% of its length

1: greater than 20% of its length

Character 167. Craniocaudal length of the acromion process of the scapula (Wilson and Sereno, 1998

0: less than one and a half times the minimum width of the scapula

blade

1: greater than one and a half times the minimum width of the scapula

blade

Character 168. Orientation of dorsal margin of the acromion process of the scapula (Novas, 1996)

0: posteroventrally, forming an acute angle with the dorsoventral axis of the scapula

1: posteriorly or subhorizontally, forming a right angle with the dorsoventral axis of the scapula

Character 169. Coracoid tubercle (Yates and Kitching, 2003)

0: absent

1: present

Character 170. Humerus/femur ratio (Gauthier, 1986)

0: subequal or shorter than 0.6

1: longer than 0.6 but shorter than 0.8

2: longer than 0.8

Character 171. Deltopectoral crest extension (Galton and Upchurch, 2004)

0: terminates less than 50% of humerus length from its proximal end

1: terminates at least 50% of humerus length from the proximal end

Character 172. Craniolateral margin of the deltopectoral crest of the humerus (Yates, 2003)

0: straight

1: strongly sinuous

Character 173. Deltopectoral crest orientation (Sereno, 1999)

0: slants at 45-60> to the transverse axis of the distal condyles

1: perpendicular to the transverse axis of the distal condyles

Character 174. Deltopectoral crest height (Wilson and Sereno, 1998)

0: tall crest with sharp edges

1: low crest with rounded edges

2: reduced to a low ridge

Character 175. Humeral distal width accounts for (Langer, 2004)

0: less or about 0.3 times the length of the humerus

1: more than 0.3 times the length of the humerus

Character 176. Well-defined, semicircular fossa on the distal flexor surface of the humerus (Yates and Kitching, 2003)

0: present

1: absent

Character 177. Proximal end of ulna (Wilson and Sereno, 1998)

0: subtriangular in outline and lacks a groove for the radius

1: triradiate because of a deep groove for reception of the radius

Character 178. Olecranon (Wilson and Sereno, 1998)

0: present as a prominent projection

1: almost completely absent

Character 179. Length of the cranial condylar process of the proximal ulna (Wilson and Sereno, 1998)

0: approximately equal to the lateral condylar process

1: much greater than the lateral condylar process

Character 180. Length of the radius (Langer, 2004)

0: less than 80% of the length of the humerus

1: greater than 80% of the length of the humerus

Character 181. Distal condyle of radius (Wilson and Sereno, 1998)

0: is subcircular or oval in outline

1: is subrectangular with a flattened caudal margin for articulation with the ulna

Character 182. Maximum linear dimensions of the ulnare and radiale (Yates, 2003)

0: exceed that of at least one of the first three distal carpals

1: less than any of the distal carpals

Character 183. First distal carpal 1 (Sereno, 1999)

0: is narrower transversely than metacarpal I

1: is subequal, or greater, in transverse width compared to metacarpal one

Character 184. Transverse width of the first distal carpal (Gauthier, 1986)

0: less than 120% of the transverse width of the second distal carpal

1: greater than 120% of the transverse width of the second distal carpal

Character 185. Distal carpal V (Sereno, 1999)

0: present

1: absent

Character 186. Proximal end of first metacarpal (Sereno, 1999)

0: flush with other metacarpals

1: inset into the carpus

Character 187. Proximal width of the first metacarpal respect to its length (Sereno, 1999)

0: less than 65% of its length

1: 65%-80% of its length

2: greater than 80% of its length

3: broader proximally than long

Character 188. Proximal width of first metacarpal respect to metacarpal II (Yates and Kitching, 2003)

0: less than the proximal width of the second metacarpal

1: greater than the proximal width of the second metacarpal

Character 189. Distal condyles of metacarpal I (Langer, 2004)

0: approximately aligned

1: the lateral condyle more distally expanded

Character 190. Metacarpal II respecto to III (Gauthier, 1986)

0: shorter or subequal

1: longer

Character 191. Extensor pits in metacarpals I-III (Langer, 2004)

0: absent, or shallow and symmetrical

1: deep and asymmetrical

Character 192. Shaft of metacarpal IV respecto to that of metacarpals I-III (Langer, 2004)

0: about the same width
1: significantly narrower

Character 193. Proximal portions of metacarpals IV and V (Gauthier, 1986)

0: set lateral to metacarpals III and IV respectively

1: set at the palmar surfaces of metacarpals III and IV respectively

Character 194. Shape of the fifth metacarpal (Yates, 2003)

0: longer than wide at the proximal end with a flat proximal surface

1: close to as wide as it is long with a large subcircular proximal articulation surface

Character 195. Length of manual phalanx I.1 (Sereno, 1999)

0: longer than metacarpal I

1: shorter than metacarpal I

Character 196. Ventrolateral twisting of the transverse axis of the distal end of the first phalanx of manual digit one relative to its proximal end (Sereno, 1999)

0: absent

1: present proximodorsal lip aligned with dorsal margin of medial distal condyle

2: present proximodorsal lip aligned with central region of medial ligament pit of the distal condyle

Character 197. Proximal heel on first phalanx on digit I (Sereno, 1999)

0: proximal heel absent

1: proximal heel present

Character 198. Length of the ungual of manual digit one (Sereno et al., 1993)

0: smaller than the length of the ungual of manual digit two

1: subequal to ungual of second digit

2: larger than second ungual

Character 199. Unguals of manual digits II and III (Gauthier, 1986)

0: slightly curved

1: strongly curved

Character 200. Manual phalanx II.2 respect to phalanx II.1, indicating the elongation of the penultimate phalanges in the manus (Gauthier, 1986)

0: shorter than phalanx 1

1: longer than phalanx 1

Character 201. Shape of non-terminal manual phalanges (Yates, 2003)

0: longer than wide

1: as long as wide

Character 202. Manual digit V (Langer, 2004)

0: possesses phalanges

1: lacks phalanges

Character 203. Convexity of dorsal margin of the ilium (Gauthier, 1986)

0: straight or sinusoidal

1: strongly convex

Character 204. Step on dorsal margin of ilium (Gauffre, 1996)

0: is smoothly convex (mildly or strongly) in lateral view

1: has a "step-like" sigmoid profile in lateral view

Character 205. Buttress between preacetabular process and the supra-acetabular crest of the ilium (Gauthier, 1986)

0: present

1: absent

Character 206. Preacetabular process of the ilium, anterior projection (Yates, 2003)

0: short, not extending cranially to the pubic peduncle

1: elongated, extending cranially to the pubic peduncle

Character 207. Shape of preacetabular process (Sereno, 1999)

0: rounded, rectangular, blunt profile

1: triangular and pointed

2: expanded dorsoventrally towards its anterior end producing a hatchet-shaped profile

Character 208. Depth of the preacetabular process of the ilium (Gauthier, 1986)

0: much less than the depth of the ilium above the acetabulum

1: subequal to the depth of the ilium above the acetabulum

Character 209. Length of the preacetabular process of the ilium (Yates and Kitching, 2003)

0: less than twice its depth

1: greater than twice its depth

Character 210. Ventral margin of iliac acetabulum (Gauthier, 1986)

0: straight

1: concave, defining a partially opened acetabulum

2: concave, defining a fully opened acetabulum

Character 211. Acetabular fossa proportions (Yates, 2003)

0: longer than tall

1: taller than long

Character 212. Supracetabular crest

0: weakly developed

1: well developed

Character 213. Supracetabular crest of the ilium at its widest point (Yates, 2003)

0: halfway between the pubic and ischial contacts

1: above the base of the pubic peduncle

Character 214. Length of the postacetabular process of the ilium (Yates and Kitching, 2003)

0: greater than 30% of the total length of the ilium

1: less than 30% of the total length of the ilium

Character 215. Shape of the caudal margin of the postacetabular process of the ilium (Yates, 2003)

0: rounded or blunty pointed

1: square ended

Character 216. Ventral margin of the postacetabular process of the ilium (Langer, 2004)

0: mainly vertical

1: slopes dorsomedially

2: bears a well developed brevis shelf

Character 217. Length of the pubic peduncle of ilium (Sereno, 1999)

0: less than twice the craniocaudal width of its distal end

1: greater than twice the craniocaudal width of its distal end

Character 218. Caudally projecting heel at the distal end of the ischial peduncle (Yates, 2003)

0: absent

1: present

Character 219. Ischial peduncle of the ilium (Galton and Upchurch, 2004)

0: is subequal in length to the pubic peduncle, giving the long-axis of the iliac blade a nearly horizontal orientation

1: is reduced so that the long-axis of the iliac blade slopes strongly craniodorsally in lateral view

Character 220. Ischiadic plate extension (Novas, 1992)

0: extended for more than half the length

1: restricted to its proximal third

Character 221. Notch separating caudoventral end of the ischial obturator plate from the ischial shaft (Yates, 2003)

0: present

1: absent

Character 222. Elongate interischial fenestra (Yates, 2003)

0: present

1: absent

Character 223. Shape of the transverse section of the ischial shaft (Sereno, 1999)

0: ovoid to subrectangular

1: triangular

Character 224. Orientation of the long axes of the transverse section of the distal ischia (Wilson and Sereno, 1998)

0: meet at an angle

1: coplanar

Character 225. Distal end of ischium respect to the rest of the shaft (Langer, 2004)

0: not expanded

1: expanded

Character 226. Length of ischium (Salgado et al., 1997)

0: less than that of the pubis

1: greater than that of the pubis

Character 227. Pelvis (Novas, 1996)

0: propubic

1: opisthopubic

Character 228. Pubis/femur length (Novas, 1996)

0: <0.5

1: >0.7

Character 229. Pubic tubercle on the lateral surface of the proximal pubis (Yates, 2003)

0: present

1: absent

Character 230. Shape of pubic tubercle (Yates, 2003)

0: low and rounded

1: enlarged, laterally projecting, semilunate plate

Character 231. Pubic apron margins in cranial view

0: lateral margins straight

1: with concave profile

Character 232. Width of the conjoined pubes (Yates and Kitching, 2003)

0: less than 75% of their length

1: greater than 75% of their length

Character 233. Minimum transverse width of the pubic apron (Yates, 2007)

0: much more than 40% of the width across the iliac peduncles of the ilium

1: less than 40% of the width across the iliac peduncles of the ilium

Character 234. Orientation of the pubic blades (Wilson and Sereno, 1998)

0: transverse

1: twisted caudomedially

2: twisted caudolaterally

Character 235. Tuberosity lateral to femoral head ligament (Novas, 1996)

0: prominent

1: reduced

Character 236. Femoral shaft (Galton and Upchurch, 2004)

0: has a sigmoid curve

1: is straight (in cranial or caudal view)

Character 237. The cranial face of the femur, in lateral view (Galton and Upchurch, 2004)

0: is convex

1: is straight

Character 238. Horizontal cross-section through the femoral shaft (Wilson and Sereno, 1998)

0: is subcircular

1: moderately elongated transversely

2: strongly elliptical

Character 239. Lesser trochanter (Galton and Upchurch, 2004)

0: is well-developed or reduced to a still visible ridge

1: is completely absent

Character 240. Shape of the lesser trochanter (Gauthier, 1986)

0: small rounded tubercle

1: elongate ridge that is oriented dorsoventrally

Character 241. Lesser trochanter height (Galton and Upchurch, 2004)

0: is a ridge-like structure, or reduced

1: is developed into a prominent sheet-like structure

Character 242. Proximal portion of the insertion of the m. iliofemoralis cranialis in the proximal femur (Langer, 2004)

0: merges with femoral shaft

1: separated from the shaft by a marked cleft

Character 243. ‘Trochanteric shelf’ (Langer, 2004)

0: absent

1: present

Character 244. Fossa trochanterica (Novas, 1996)

0: absent

1: present

Character 245. Height of the fourth trochanter (Gauthier, 1986)

0: tall crest

1: low rugose ridge

Character 246. Distal end of fourth trochanter (Galton and Upchurch, 2004)

0: lies above mid-shaft length

1: lies at or below mid-shaft length

Character 247. Profile of the fourth trochanter of the femur (Langer, 2004)

0: rounded and symmetrical

1: asymmetrical with a steeper distal slope than the proximal slope and distinct corners

Character 248. Fourth trochanter mediolateral position on femur (Yates, 2007)

0: lies close to the midline of the caudal surface of the shaft

1: lies close to the caudomedial margin of the shaft

Character 249. Extensor depression on the distal femur (Yates and Kitching, 2003)

0: absent

1: present

Character 250. Lateral condyle of tibia (Yates and Kitching, 2003)

0: set on the center of its medioproximal corner

1: level with the medial condyle at the caudal border of the bone

Character 251. Cnemial crest (Novas, 1996)

0: absent

1: present

Character 252. Cnemial crest on the tibia (Wilson and Sereno, 1998)

0: is directed cranially

1: is directed laterally

Character 253. Distal end of tibia (Novas, 1996)

0: anteroposteriorly elongated

1: subquadrangular

2: transversely elongated

Character 254. Mediocranial corner of distal tibia forms (Langer, 2004)

0: rounded obtuse or right angle

1: acute angle

Character 255. Lateral surface of distal tibia (Novas, 1996)

0: smooth

1: with longitudinal groove

Character 256. Caudomedial notch in distal tibia (with respective bump in the proximal astragalus) (Langer, 2004)

0: absent

1: present

Character 257. Posterior descending process of the distal tibia (Wilson and Sereno, 1998)

0: exceeds laterally the craniolateral corner of the distal tibia

1: protrudes laterally as far as the craniolateral corner of the distal tibia

2: set well back from the craniolateral corner of the distal tibia

Character 258. Trigonal striated articular crest on medial surface of the proximal end of the fibula (Wilson and Sereno, 1998)

0: absent

1: present

Character 259. Muscle scar trochanter on the lateral surface of fibula (at mid-length) (Wilson and Sereno, 1998)

0: absent

1: present

Character 260. Fibula respect to tibia at the middle of their shafts (Langer, 2004)

0: wider than half the width of the tibia

1: subequal or narrower than half the width of the tibia

Character 261. Ascending process of astragalus (Novas, 1996)

0: narrow ridge

1: pyramid-shaped

Character 262. Depression and vascular foramina in front of the base of the astragalar ascending process (Wilson and Sereno, 1998)

0: present

1: absent

Character 263. Dorsally facing horizontal shelf forming part of the fibular facet of the astragalus (Sereno, 1999)

0: present

1: absent with a largely vertical fibular facet

Character 264. Shape of the caudomedial margin of the astragalus in dorsal view (Yates and Kitching, 2003)

0: forming a moderately sharp corner of a subrectangular astragalus

1: evenly rounded without the formation of a caudomedial corner

Character 265. Ossified distal tarsals (Gauthier, 1986)

0: two or more

1: absent

Character 266. Metatarsal III length-tibia length ratio (Wilson and Sereno, 1998)

0: 0.4 or higher

1: less than 0.4

Character 267. Width of the midshaft of the first metatarsal (Wilson and Sereno, 1998)

0: not greater than the width of the midshaft of the second metatarsal

1: greater than the width of the midshaft of the second metatarsal

Character 268. Shape of the medial margin of the proximal surface of the second metatarsal (Sereno, 1999)

0: straight

1: concave

Character 269. Shape of the lateral margin of the proximal surface of the second metatarsal (Sereno, 1999)

0: straight

1: concave

Character 270. Transverse width of the proximal end of the fourth metatarsal (Sereno, 1999)

0: less than twice the craniocaudal depth of the proximal end

1: at least twice the craniocaudal depth of the proximal end

Character 271. Distal articular surface of metatarsal IV

0: broader than deep

1: as broad as deep

2: deeper than broad

Character 272. Metatarsal V length (Wilson and Sereno, 1998)

0: is less than 50% of metatarsal III length

1: is at least 75% of metatarsal III length

Character 273. Metatarsal V shape (Galton and Upchurch, 2004)

0: proximal and distal ends subequal in breadth

1: triangular shaped, with wide proximal surface and pointed distal end

Character 274. At least some pedal phalanges, apart from unguals (Wilson and Sereno, 1998)

0: are longer proximodistally than their transverse widths

1: are wider transversely than their proximodistal lengths

Character 275. Ungual on pedal digit I (Wilson and Sereno, 1998; Galton and Upchurch, 2004)

0: is shorter than other pedal unguals

1: subequal than other pedal unguals

2: is longer than other pedal unguals

Character 276. Shape of the ungual of pedal digit one (Yates and Kitching, 2003)

0: shallow, pointed, with convex sides and a broad ventral surface

1: deep, abruptly tapering, with flattened sides and a narrow ventral surface

Character 277. Number of phalanges in pedal digit four (Yates and Kitching, 2003)

0: four

1: fewer than four

**1.b Data matrix.** The phylogenetic dataset is included here. Character scorings enclosed in brackets represent polymorphic scorings (due to ambiguity or true polymorphism). An electronic version in nexus format is uploaded as supplementary information.

Lagerpeton chanarensis

???????????????????????????????????????????????????????????????????????????????????????????????????????????????????????????????????00???????????00??????????????????????????????????????????????????????????????????0???011000?001??0?00??01???00?0????0?0?0?????0??????????????????

Marasuchus lilloensis

0???????00????0?00??????0?0?????????????????????????????????????????????????????????????0???0?0000??00?00000??00010000??000000?000000?000?0000?0000000000????????00???1000?0?00010?????????????????????00?000000000000000110000001??0?000000??0110000?01?10101??00?00??????0000???0?

Pseudolagosuchus major

????????????????????????????????????????????????????????????????????????????????????????????????????????????????????00??000000?0?0000?000?0000??00??????????????????????????????????????????????????????????????????????????????1???0??0???0??011??????1?1?1?1??01?00???????????????

Silesaurus opolensis

00???0??0???????????????????????????????????????????????????????????????????00010???????????0?0121??0??00?????????????????????????????000???????????2????????????00????0000??0??10????????????????????????000000?000100011????00101000010000??0110000?11?10100??0[01]?0????????000???10

cf.Azendhosaurus_madagascar

?0??????????????????????????????????????????????????????????????????????????00010???????00??0?01211?0?000?????????????????????????????????????????????????????????????????????????????????????????????????????????????????????????????????????????????????????????????????????????10

Azendhosaurus laroussi

?0??????????????0???????????????????????????????????????????????????????????00010???????00??0?01211?01000?????????????????????????????????????????????????????????????????????????????????????????????????????0???????????????????????????????????????????????????????????????????10

Pisanosaurus mertii

?0????????????????????????????????????????????????????????????????????????????10?1?0???100?00?1121??0100????????????????????????????????????????00??2??1??????????????1??????????????????????????????????????120?0?0???????????011????????00??????????11?1110???1??????????1??????00

Heterodontosaurus tucki

00?000??0[01]1?0?00?0?????0101000?01??000???????001000?0000??0????0????????????0010?1000??100?0001121??0100?000???00?0010??000000?000000?000?0000?0000?2?01?????????00???1000?0?0?000??000?000000?100000?000111012000001100???0000111??01010000??11?000??01?21?????1??0???????0??????0?

Lesothosaurus diagnosticus

00?000??00?00?00?0?????010100000??0000?1???0?00?0?0?00000?0?00?0??????????0?001001?00??100100011211?0100????????????????????????????0????????????0??2??1??????????????1?????????0????????????????????????????12?????1???0??0000111??01010000??101?0?0?00?21010??1??????????0??????00

Guaibasaurus candelariens

????????????????????????????????????????????????????????????????????????????????????????????????????????????????????????????????????0?000????????0???????????????10???????????????????????????????????????????0010011?001??1?10010110?0100000?00?0000????21110??01000??????0?000?0??

Eoraptor lunensis

00?000??0111100001?0???1010010?11?0001?1??0010????001?000?1?????????????????000000?0???100?00000100000000???????????11????0100?010000?000????1?000?01?10?????????10???000??0????1????????10110????000010??0??020100?[12]?101??0?0?011??0?0??000??00?00???1??2010???0??????????0??????00

Herrerasaurus ischigualastensis

0000000001?010000000000011000000?00101000?0010000000100100?00000000000000010000000?10000000000000000000001110010?000110000010?00?00000000000010?00000010000010000101100000000000101001000001110?01011010?0000020100000001?0100?[01]10??002100000?0110010011?1010100010000000010??000?0?

Coelophysis bauri

00000000011110020000000101001001000001?11?000000000010010011???00??0000000100000001100000000000000000000011110100?001100002101101000000000000101?00?2011?00010???0000000000000000010?100011111?001?1101001121020100120?01??001?01???010?00000?0??00?0??1???110??1??0?000?001?000000?

Liliensternus liliensterni

????????????????????????????????????????????????????????????????????????????????????????????????????????????10?000001100002101101000000000000101200?2?11?0?010100000?0000000000000?????????????0???????001121020100120101000010011??010100000?0?1000001??11110?01100???????1?000?00?

Elaphrosaurus bambergi

???????????????????????????????????????????????????????????????????????????????????????????????????????????????00?001100002101?01000000000000101000?2?11?0???????000??000000?00?????1??????????????????001121020100120101000010011??010?00000?0??00??0?????0?0??1?????????????0?????

Saturnalia tupiniquim

1???????????????????000????????1?????????????????????????0???????00?0000????00?11???????00?0?00121?000000??????0?1??11???????1?01000000?0100010000000?10010?0????00110100101000000?????????????????????0?001001010002100111001001010000?00000?011001000111010100010000000011000??000

Pantydraco caducus

10?000??0??0??1??2???????0?10001?00001?11?00??000?0??????00100?0000100010?01000111?00000000111012110000000?11?20?1??110000010110100000000100010100?001?101100000?000001001010000?01????????????????????0010100201?01?100111?0??????0??0?00001000?001?00??111?10001010000111001010000

Thecodontosaurus antiquus

10??0???0?????????????????????????????????????????????????????????????????0?000111?????000?11101211000000??????????????????????0?0?000??010001??00?001????1?0????00000100?010000???01?00111?0100111000?????????????????????0????????????0000100??0010??????1?????1?????0??????????00

Efraasia minor

1010000?0100??110211???010?????1000101?11?????000??10????0?0?????00100110?01000101??11?100011101?11000000????120?100111000011?1010?000000?00010?000011??01000101?0000011010100000011???111????00111??0?0010100201101210?11110100?010000?00001000?001000?0211?10001?100001?1?01010000

Plateosaurus engelhardti

101000000100111102111101100111110001010110100001000100110110000011010110110000020110111100111101211000000011012011001110000111101000020001000101001101110000011100000011010100000011111111100111111000000101002011001110100101001000000100001000?001010?02110100010100001?1001010001

Plateosaurus gracilis

1010000?0100??110211110110?1?1?1?00101?11????00?0??????1?1????????????1?????000?01?0???100111?01211?00000??????0???????0???11?10?0?00?000??0010?00?10???0???0????000?0110?01000?????????????????????????????????1???1110?00101001000000?00001000?00101?1?2?1????????0??????????1??0?

Ruehleia bedheimensis

?????????????????????????????????????????????????????????????????????????????0?????????????????????????????????0?1??????00011??0?0?00?000??0010?00?01?1?00???????000?0110101000?0??????????????????????00101002?1100?110???????????0000?00001000?00101?1???1????????????????????????

Coloradisaurus brevis

1010010?0110?1121211110110?1?111?101011111?001?00001001101?????0110111101110000201?01?1100011101211000000??????00100???00001210010000?000100010?00???????010?????0001011110100?????????????????????????0??01002011???110?11?01?0?1?1000100001000?0011101020111???1010?00111001020001

Massospondylus carinatus

101001010110?11212011100101?111111010101100011110101000201??0110110110001110000201?00?11000111[01]1211100000??10?200100111000011?00100002000100010?001011110000?011?000101101010000001111121110?11121200?0001010020110011001111010011?1000?00001000?00101?102?1?10001010000111001020001

Lufengosaurus huenei

10????01?????1?202111100101??11101110111101011?10?01001?0?100??01101??00??00000??1101?1100011101211?000?001???200100111?00011100?0000?000100010?000011??000?1111000010111101000000111?12111??111212001?001010020110011001111010011?1000?00001000?00111?102?1?10001?1000011??01020001

Gyposaurus sinensis

1???010?0110??1?????????????????????????????????????????????????????????????0002????????00?11?01211?00??0??????0010011??00011?00?0?00?00010001??00?01???0????????000?0111001?000??????1211???111212??1?00101002011001100???1010011?1??0??00???0??001?????2?1?????1?1?????????1????0?

Yunnanosaurus huangi

1??10?0101?0??12?2?1????101?01?111??01?11?1011??000?0?020?????????????????1?000201?00??100011101211?00??0?????200100????00011100?0?00?000100010?00?01?1?0????????00010111101?0000??????2111????1212??1?001010020110011001111010011?1000?00001000?00111?102?1?1???1010?001???01????01

Riojasaurus incertus

10?000?101?0?11??2?1????101????100?101011??01111000?00010?????????????10?100000101?00??0001?1?01211000??0?1??1100100110?0001011010000100010001[0 1]?00101?1?0000110??000001111011000001?1??1111?0111112001?01101002111002110101101?011?0000110101100?01111?102?101??01010?00111001010?01

Anchisaurus polyzelus

10??000101?0??1?02111??0?011???111?1110110010[12]?1[01]1010?0????00???1210120001?1000201?00?011?101?01211100?10??10020010011100001??00?0000?000100010?00?01???0????????001??110100100000????11111?0?11112??0?00111012011001100?11011?011?0010?10001000?00?11?1?2?1?[12]?0?1??0000????0101000?

Aardonyx celestae

11?000010?1?11?20211100010?????????????1??10???10?01????????????1?????0?????0002?0??????0011???1211100010??????0?1??1101????1?00?001000?0100?10?00?0111?0100110??????0???????000?0????13?1?0????1??0?1??????????????????101101?0?1?0100?100011???01100???211?20??????0??111???1?0?01

Leonerasaurus taquetrensis

????????????????????????????????????????????????????????????????????????????000?0????????1??1??1211100110?????20010011?100011100?[01]2000000100010?00?111110????????00?0??10[01]110????????????????????????????111012011???100???[01]0?????????????0?????????????????????????????11????0?0?0?

Jingshanosaurus xinwaensis

1??00??10?10??1?02???????????0?????????1??????????0?????0???????????????????00010???0???00?11?012???00??0??????0???????????????0?0?00?000???????00?01???1????????0000?110?010?0000??????11?????11??????0010100201110?100??1?01?0?1?1000?10[0 1]01?00?0[0 1]0????0??1???????1???0?????1????01

Camelotia borealis

????????????????????????????????????????????????????????????????????????????????????????????????????????????????????????000????0?0??0?000????1??00??????????????????????????????????????????????????????????????????????????????????????10101100?01?1???????????????????????????????

Melanorosaurus readi

1??01[0 1]010110??1?02?1???1101????111??01?11010?11???0??0020???011???????????1?00010??00??1000???01211100?10??????00100????0001??10?0?000000100011?00?1211?0000110??0100?110010110100?????[23]?1?????11??????011[01]10[01]2011001110???10??011?0000?1?101100?010110102?1?20???010?00111?01????0?

Antetonitrus ingenipes

???????????????????????????????????????????????????????????????????????????????????????????????????????????????0????????????0?10???110000101111?00???????1?0110??2100?200010110100????1311?????111?????0???????0????????????????????000?10201100?01111??02?1?200??????1111??01??0???

Lessemsaurus sauropoides

???????????????????????????????????????????????????????????????????????????????????????????????????????????????01?11????000?0?10?11110000102?11?10?????????01?1??2100??00?101101?0????1311?????111?????00101002010101100???????0?1?1000110101100?011110102?10??????10???11??0101????

Blikanasaurus cromptoni

???????????????????????????????????????????????????????????????????????????????????????????????????????????????????????????????????????????????????????????????????????????????????????????????????????????????????????????????????????????????????????????????0??110011111?011100??

Vulcanodon karibaensis

??????????????????????????????????????????????????????????????????????????????????????????????????????????????????????????????1????????????????????1????0??1??01??1?1?20002011?111?????????????????????????????010???1011110011?????010?10201000?11?1???02?1???0??111111101?11011???

Kotasaurus yamanpalliensi

????????????????????????????????????????????????????????????????????????????????????????????????????1012????10?0?0??????????0?01?????0???10?211?2[01]?11????00111???0000??0001011?11??????????????????????0??11012?10[01]01101?11???10?1??0???11201000?110??????????10??1???11????????????

Isanosaurus attavipachi

??????????????????????????????????????????????????????????????????????????????????????????????????????????????????????????????01???????????1011?2[01]??????????????1001?????????????????????????????????????????????????????????????????????121???0?10011??????????????????????????????

Barapasaurus tagorei

???????????????????????????????????????????????????????????????????????????????????????????????????????????????0?0??????????1????????0???012211?21?110??10011?0???101?????2??11??1??????????????????????????102010???1?1???00?1??1??111??121???0?11?????1?????11???1????????????????

Shunosaurus lii

11001?111??0?112?2000000101?00???11?11010??0?2?00?00?10211??01112210?20000?1110100?00?0011?11001211110121???0141?0??1111???10?01?????00??1?22???20011???0001110?100??12000211?1101100?011?1???0?0?2??1?10111102010101101?11?011011?1111?1121???0?1100???1??1???1??11?111100?1?121101

Omeisaurus maoianus

110011111?10?112020100101?1000?1?11111010???02??1??0?10211?????122100???00?1110100?0??0111?0100121?110121???0041?0??1111???12?01?????00??012211?31?120??1001110110011120002011?101????00?1???00???2????10112102010101101?1100?1011?1111?112????0?1100???1??1??11??1???11100?1?121101

Lapparentosaurus madagascariensis

????????????????????????????????????????????????????????????????????????????????????????????????????????????????????????0????????111??0?1002?1??????????????????????????????????????????????????????????????????????????????????????????????????????????????????????????????????????

Volkheimeria chubutiensis

????????????????????????????????????????????????????????????????????????????????????????????????????????????????????????0????????1111?0?0002?11?????????????????????????????????????????????????????????????????????????????????????????????????????????????????????????????????????

Patagosaurus fariasi

???????????????????????????????????????????????????????????????????????????????????????????????1????????????????2?22111?101111?1?2212?0110?2?11?31?1????1?0?1????001112000201111?1?????????????????????10112102?10101101???00110?1?111111121???0?110????02?1????????????????????????

Ferganasaurus verzilini

?????????????????????????0???????????????????2????????????????????????????????????????????????????????????????????????????????????????????????????????????????????????200020111101????000110?001?02??1?101121020101?1101??????1011??11111121???0?1101???1??1??????111???????????????

Diplodocus

1100111111?01112020?0??0101?0001?1111111000102101010110211?011112210020?0011110100?00?011100100121??101210?1003020221111?1211111122120021012211?32012???10011101?00111200020111111?011000?1??001002??1?10112102?101011011??101?010??111?1121???0?110????1??1??11???11?1?????1?12?101

Camarasaurus

1100111111?011120201101010100001?11111010001021010100102[01]11011112210020?00111101?0100?011100100121??10121???003020221111112111?1112120111012211?330120??1?011??10001112000201111111???000110?0?1?02????101121020101011011110101011?111111121???0?110?1?112?1?211??111111????1?121101

Brachiosaurus

1100111111101112020110101?100001?111111100010?101010010211101111221002?00011110100?00?011100100121??10121???0040?0221111?12121?1122120111012211?330120??10011????00111200020111111???????01??001002??1?10112102?101011011??0101011??11111121???0?110????1??1???????1??11000?1?12?10?

**1.c List of taxa and specimens examined.** The taxon sampling regime used in the phylogenetic analysis a large number of outgroup taxa (14 non-sauropodomorph taxa) that were included to test more thoroughly the relationships of basal dinosaurs and to infer the plesiomorphic state at the base of Sauropodomorpha. The sampling of sauropodomorph dinosaurs (‘prosauropods’ and sauropods) includes twenty basal sauropodomorphs that were selected because of the lack of current consensus on the phylogenetic relationships of these taxa (i.e., the monophyly of Prosauropoda and their interrelationships). Regarding the sampling of sauropods, special focus was paid to select basal sauropods (and eusauropods) so that the plesiomorphic condition and the origins of sauropods could be evaluated. Some derived eusauropods were also included to extend the taxon sampling up to the basal nodes of Neosauropoda.

The following list details the taxa studied for the phylogenetic analysis (in alphabetical order). Specimen numbers are detailed for those taxa that were first-hand revised. The taxa that were not first hand revised include the reference from which the data was taken.

*Aardonyx celestae :* Yates et al. (2010)

*Anchisaurus polyzelus :* YPM 1883 YPM 208, YPM 209, AM 41/109, AM 41/118

*Antetonitrus ingenipes :* BPI.I.4952

*Azendhosaurus laroussi :* MNHN ALM-351, MNHN ALM-353, MNHN ALM-355, MNHN ALM-365, MNHN ALM-424

*cf.Azendhosaurus :* UA 10603, UA 10604

*Barapasaurus tagorei :* Jain et al. (1975, 1979), Bandyopadhyay et al. (2010)

*Blikanasaurus cromptoni :* SAM K403

*Brachiosaurus :* Wilson (2002)

*Camarasaurus :* CM 11383, Wilson (2002)

*Camelotia borealis :* Galton (1998)

*Coelophysis bauri :* MCZ 4326, MCZ 4779, MCZ 4333, MCZ 4327, MCZ 4334, AMNH 2722, AMNH 2750, AMNH 7223, AMNH 7241, CM s/n

*Coloradisaurus brevis :* PVL 3967, PVL n.6

*Diplodocus :* Wilson (2002)

*Efraasia minor :* SMNS 11838, SMNS 12216, SMNS 12354, SMNS 12667, SMNS 12668, SMNS 12684, SMNS 14881, SMNS 17928

*Elaphrosaurus bambergi :* MB specimen mounted in exhibit

*Eoraptor lunensis :* PVSJ 512

*Ferganasaurus verzilini :* Alifanov and Averianov (2003)

*Guaibasaurus candelariensis :* MCN 2355, MCN 2356

*Gyposaurus sinensis :* NGMJ V 108

*Herrerasaurus ischigualastensis :* PVL 2559; PVSJ 373; MCZ 7063, MCZ 7064

*Heterodontosaurus tucki :* SAM K1332, SAM K337, SAM K8034

*Isanosaurus attavipachi :* Buffetaut et al. (2000)

*Jingshanosaurus xinwaensis :* Zhang and Yang, 1994

*Kotasaurus yamanpalliensis :* Yadagiri, 2001

*Lagerpeton chanarensis :* PVL 4619, PVL 4625, MCZ 4121

*Lapparentosaurus madagascariensis :* MNHN MAA-91-92

*Leonerasaurus taquetrensis :* MPEF-PV 1663

*Lesothosaurus diagnosticus:* MNHN-LES 18

*Lessemsaurus sauropoides :* PVL 4822

*Liliensternus liliensterni :* MB R.2175

*Lufengosaurus huenei :* IVPP V 15

*Marasuchus lilloensis :* PVL 3870, PVL 3871, PVL 3872, PVL 4672

*Massospondylus carinatus :* SAM K7904, SAM K1325, BPI.I.4376, BPI.I.4779, BPI.I.4930, BPI.I.4934, BPI.I.5241, MNHN s_n, SAM K388, SAM K391, SAM K398, SAM K2352, SAM K2768, SAM K2770, SAM K2772, SAM K3358, SAM K3451, SAM K5132, SAM K5133, SAM K5134, SAM K5135, SAM K10654, BPI.I.2714, BPI.I.4243, BPI.I.4377, BPI.I.4693, BPI.I.4789, BPI.I.4860, BPI.I.4924, BPI.I.4945, BPI.I.5143, BPI.I.5238, BPI.I.5253, BPI.I.6276

*Melanorosaurus readi :* BPI.I.5355, SAM K3449, SAM K3450, NMQR 1551, NMQR 3314

*Omeisaurus maoianus :* Tang et al. (2001)

*Patagosaurus fariasi :* PVL 4170, MACN-CH 933, MPEF-PV 1670

*Pisanosaurus mertii :* PVL 2577

*Plateosaurus engelhardti :* BSP 1962.I.153, BSP 1962.XLVI.#, UEN 552, UEN #, MB skelett 18, MB R.1937 (skelett 24), MB.I.121 (skellet 25), MB skellet 45, AMNH 6810, MB A58, MB skelett 1, MB skelett 30a, MB skelett 37, MB skelett 42, MB skelett 44, MB skelett C, GPIT 2, GPIT 3, GPIT 1, GPIT B, SMNS F65, SMNS 13200, SMNS 12950, SMNS 54935, SMNS 81914, SMNS 12949, SMNS F5, SMNS F20, SMNS F37

*Plateosaurus gracilis :* SMNS 5715, GPIT 18318a, GPIT Aixheim, GPIT 18392

*Pseudolagosuchus major:* PVL 4629, MLR 53

*Riojasaurus incertus :* PVL 3808, MLR 56, PVL 3806, PVL 3805, PVL 3815, PVL 3844, PVL 3845, PVL 3846, PVL 3847, PVL 3662, PVL 3663, PVL 3668, PVL 3669, PVL 3526, PVL 3533, PVL 3467, PVL 3472, PVL 3478, PVL 3392, PVL 3393

*Ruehleia bedheimensis :* MB RvL 1, MB RvL 2, MB RvL 3

*Saturnalia tupiniquim :* MCP 3844, MCP 3845

*Shunosaurus lii :* Zhang (1988), Chatterjee and Zheng (2002)

*Silesaurus opolensis :* Dzik (2003)

*Thecodontosaurus antiquus :* YPM 2192, YPM 2193, YPM 2194, YPM 2195, and Benton et al. (2000) for BRSMG-ANSP material

*Pantydraco caducus :* Yates (2003)

*Volkheimeria chubutiensis :* PVL 4077

*Vulcanodon karibaensis :* Raath (1972) and Cooper (1984)

*Yunnanosaurus huangi :* NGMJ 6004546 (after Barrett et al. 2007)

**1.d Strict consensus tree and list of synapomorphies.** The complete strict consensus (with node numbers) is included followed by the list of unambiguous synapomorphies that diagnose each of the nodes present in the strict consensus. Synapomorphic characters common to all MPTs are given first, followed by features that diagnose each node in only some of the MPTs.

The following list details the unambiguous synapomorphies present in the MPTs for the nodes of the strict consensus. MDD refers to “more derived dinosauromorphs”.

Node 52 (*Pseudolagosuchus* + MDD):

All trees:

Char. 228: 0 --> 1

Char. 261: 0 --> 1

Node 53 (*Azhendosaurus* + *Silesaurus*):

All trees:

Char. 107: 0 --> 1

Some trees:

Char. 210: 12 --> 0

Node 54 (Dinosauria):

All trees:

Char. 119: 0 --> 1

Char. 235: 0 --> 1

Node 55 (Ornithischia):

All trees:

Char. 95: 0 --> 1

Char. 209: 0 --> 1

Char. 254: 0 --> 1

Char. 260: 0 --> 1

Some trees:

Char. 79: 0 --> 1

Char. 103: 0 --> 1

Char. 155: 0 --> 1

Node 56 (*Lesothosaurus* + *Heterodontosaurus*):

All trees:

Char. 227: 0 --> 1

Char. 253: 1 --> 2

Node 57 (*Guaibasaurus* + Neotheropoda):

All trees:

Char. 225: 0 --> 1

Char. 254: 0 --> 1

Char. 256: 0 --> 1

Some trees:

Char. 215: 0 --> 1

Char. 257: 1 --> 0

Node 58 (Theropoda):

All trees:

Char. 100: 1 --> 0

Char. 165: 0 --> 1

Some trees:

Char. 26: 0 --> 1

Char. 53: 0 --> 1

Char. 75: 0 --> 1

Char. 82: 1 --> 0

Char. 96: 1 --> 0

Char. 97: 2 --> 01

Char. 99: 1 --> 0

Char. 108: 0 --> 1

Char. 116: 1 --> 0

Char. 170: 1 --> 0

Char. 191: 0 --> 1

Char. 192: 0 --> 1

Char. 202: 0 --> 1

Char. 222: 1 --> 0

Node 59 (Neotheropoda):

All trees:

Char. 165: 1 --> 0

Char. 260: 0 --> 1

Some trees:

Char. 216: 1 --> 2

Char. 218: 0 --> 1

Char. 229: 0 --> 1

Char. 271: 0 --> 1

Node 60 (Sauropodomorpha):

All trees:

Char. 1: 0 --> 1

Char. 173: 0 --> 1

Some trees:

Char. 175: 0 --> 1

Char. 180: 1 --> 0

Char. 207: 0 --> 1

Char. 217: 0 --> 1

Char. 225: 0 --> 1

Char. 247: 0 --> 1

Node 61 (*Thecodontosaurus* + *Pantydraco*):

All trees:

Char. 158: 0 --> 1

Some trees:

Char. 88: 1 --> 0

Node 62 (*Thecodontosaurus* + MDD):

All trees:

Char. 92: 0 --> 1

Char. 94: 0 --> 1

Char. 146: 0 --> 1

Char. 155: 0 --> 1

Char. 240: 0 --> 1

Char. 254: 0 --> 1

Char. 263: 0 --> 1

Char. 268: 0 --> 1

Char. 269: 0 --> 1

Char. 273: 0 --> 1

Some trees:

Char. 243: 1 --> 0

Node 63 (*Efraasia* + MDD):

All trees:

Char. 36: 0 --> 1

Char. 71: 0 --> 1

Char. 86: 0 --> 1

Char. 121: 0 --> 1

Char. 127: 0 --> 1

Char. 161: 0 --> 1

Char. 163: 0 --> 1

Char. 171: 0 --> 1

Char. 183: 0 --> 1

Char. 187: 0 --> 1

Char. 223: 0 --> 1

Char. 253: 1 --> 2

Some trees:

Char. 151: 0 --> 1

Node 64 (*Plateosaurus*):

All trees:

Char. 150: 0 --> 1

Some trees:

Char. 24: 0 --> 1

Char. 30: 0 --> 1

Char. 151: 1 --> 0

Char. 222: 1 --> 0

Node 65 (*Plateosaurus* + MDD):

All trees:

Char. 157: 1 --> 0

Char. 249: 0 --> 1

Some trees:

Char. 48: 0 --> 1

Char. 58: 0 --> 1

Char. 66: 0 --> 1

Char. 91: 0 --> 1

Char. 98: 0 --> 1

Char. 136: 0 --> 1

Char. 194: 0 --> 1

Char. 195: 0 --> 1

Char. 218: 0 --> 1

Node 66 (*Lufengosaurus, Coloradisaurus, Yunnanosaurus, Gyposaurus*):

All trees:

Char. 172: 0 --> 1

Node 67 (Massospondylidae):

All trees:

Char. 168: 0 --> 1

Char. 196: 1 --> 2

Some trees:

Char. 6: 0 --> 1

Char. 30: 0 --> 1

Char. 91: 1 --> 0

Char. 162: 0 --> 1

Char. 231: 0 --> 1

Char. 236: 1 --> 0

Char. 275: 1 --> 2

Node 68 (Massopoda):

Some trees:

Char. 8: 0 --> 1

Char. 27: 0 --> 1

Char. 46: 0 --> 1

Char. 47: 0 --> 1

Char. 160: 0 --> 1

Char. 198: 1 --> 2

Char. 201: 0 --> 1

Char. 229: 0 --> 1

Char. 236: 0 --> 1

Char. 248: 0 --> 1

Node 69 (*Vulcanodon* + MDD):

All trees:

Char. 264: 0 --> 1

Char. 269: 1 --> 0

Char. 272: 0 --> 1

Char. 276: 0 --> 1

Some trees:

Char. 159: 0 --> 1

Char. 181: 0 --> 1

Char. 219: 0 --> 1

Char. 233: 0 --> 1

Char. 241: 01 --> 0

Char. 245: 0 --> 1

Char. 265: 0 --> 1

Node 70 (Eusauropoda):

All trees:

Char. 129: 1 --> 0

Char. 166: 1 --> 0

Char. 237: 0 --> 1

Node 71 (*Patagosaurus* + MDD):

Some trees:

Char. 140: 1 --> 0

Char. 187: 1 --> 0

Char. 188: 1 --> 0

Char. 207: 1 --> 2

Node 72 (*Shunosaurus* + MDD):

All trees:

Char. 203: 0 --> 1

Char. 208: 0 --> 1

Char. 232: 0 --> 1

Char. 239: 0 --> 1

Char. 259: 0 --> 1

Node 73 (Neosauropoda):

Some trees:

Char. 124: 0 --> 1

Char. 125: 1 --> 2

Char. 148: 1 --> 2

Char. 180: 0 --> 1

Node 74 (Macronaria):

All trees:

Char. 21: 0 --> 1

Char. 137: 0 --> 1

Char. 148: 2 --> 3

Char. 224: 0 --> 1

Char. 225: 1 --> 0

**1.e Reduced strict consensus and additional list of synapomorphies.** The reduced strict consensus (excluding *Jingshanosaurus*, *Camelotia*, *Blikanasaurus*, and *Ferganasaurus*) provides a better resolution of basal sauropods and closely related forms (i.e., basal anchisaurian sauropodomorphs). The relationships depicted here are identical in all the MPTs (when the position of the above mentioned unstable taxa is ignored). The reduced consensus tree (with node numbers) is included followed by the list of unambiguous synapomorphies that diagnose each of the nodes present in this tree (that are absent in the complete strict consensus). Synapomorphic characters common to all MPTs are given first, followed by features that diagnose each node in only some of the MPTs.

List of unambiguous synapomorphies of nodes resolved in the reduced consensus:

Node 68 (Massospondylidae + Anchisauria):

All trees:

Char. 33: 0 --> 1

Char. 34: 0 --> 1

Char. 55: 1 --> 2

Char. 71: 1 --> 0

Char. 75: 0 --> 1

Char. 129: 1 --> 0

Some trees:

Char. 218: 1 --> 0

Node 70 (Anchisauria):

All trees:

Char. 45: 1 --> 0

Char. 66: 1 --> 2

Char. 67: 0 --> 1

Char. 68: 1 --> 0

Char. 70: 01 --> 2

Char. 76: 0 --> 1

Char. 105: 0 --> 1

Char. 206: 0 --> 1

Node 71 (*Aardonyx* + MDD):

All trees:

Char. 2: 0 --> 1

Char. 82: 1 --> 0

Char. 122: 0 --> 1

Char. 133: 0 --> 1

Char. 241: 0 --> 1

Char. 246: 0 --> 1

Node 72 (*Leonerasaurus* + MDD):

All trees:

Char. 104: 0 --> 1

Char. 150: 0 --> 1

Node 73 (*Melanorosaurus* + MDD):

All trees:

Char. 129: 0 --> 1

Char. 145: 0 --> 1

Char. 166: 0 --> 1

Node 74 (*Lessemsaurus* + *Antetonitrus*):

All trees:

Char. 166: 0 --> 2

Node 75 (Sauropoda):

All trees:

Char. 117: 0 --> 1

Char. 118: 0 --> 1

Char. 132: 0 --> 1

Char. 134: 0 --> 1

Char. 135: 0 --> 1

Char. 142: 0 --> 2

Char. 143: 0 --> 1

Char. 170: 1 --> 2

Char. 171: 1 --> 0

Char. 213: 1 --> 0

Char. 214: 0 --> 1

Char. 231: 0 --> 1

Char. 266: 0 --> 1

Char. 267: 0 --> 1

Node 78 (*Isanosaurus* + MDD):

All trees:

Char. 167: 0 --> 1

Node 79 (*Barapasaurus* + MDD):

All trees:

Char. 141: 0 --> 1

Node 80 (*Lapparentosaurus* + MDD):

All trees:

Char. 139: 0 --> 1

Node 83 (*Patagosaurus* + MDD):

All trees:

Char. 147: 2 --> 3

**1.f Support Measures.** The following tree represents the resampling-based support measures obtained in bootstrap and jackknife 1000 replicates in TNT. The alternative positions of the highly unstable taxa *Jingshanosaurus*, *Camelotia*, and *Blikanasaurus* were ignored in the trees found in the bootstrap and jackknife analyses. The values shown at the nodes represent the support measures in the following order: standard absolute bootstrap frequencies, standard absolute jackknife frequencies, GC bootstrap frequencies, and GC jackknife frequencies (see Goloboff et al. 2003 for an explanation on the use of the GC frequencies).

**
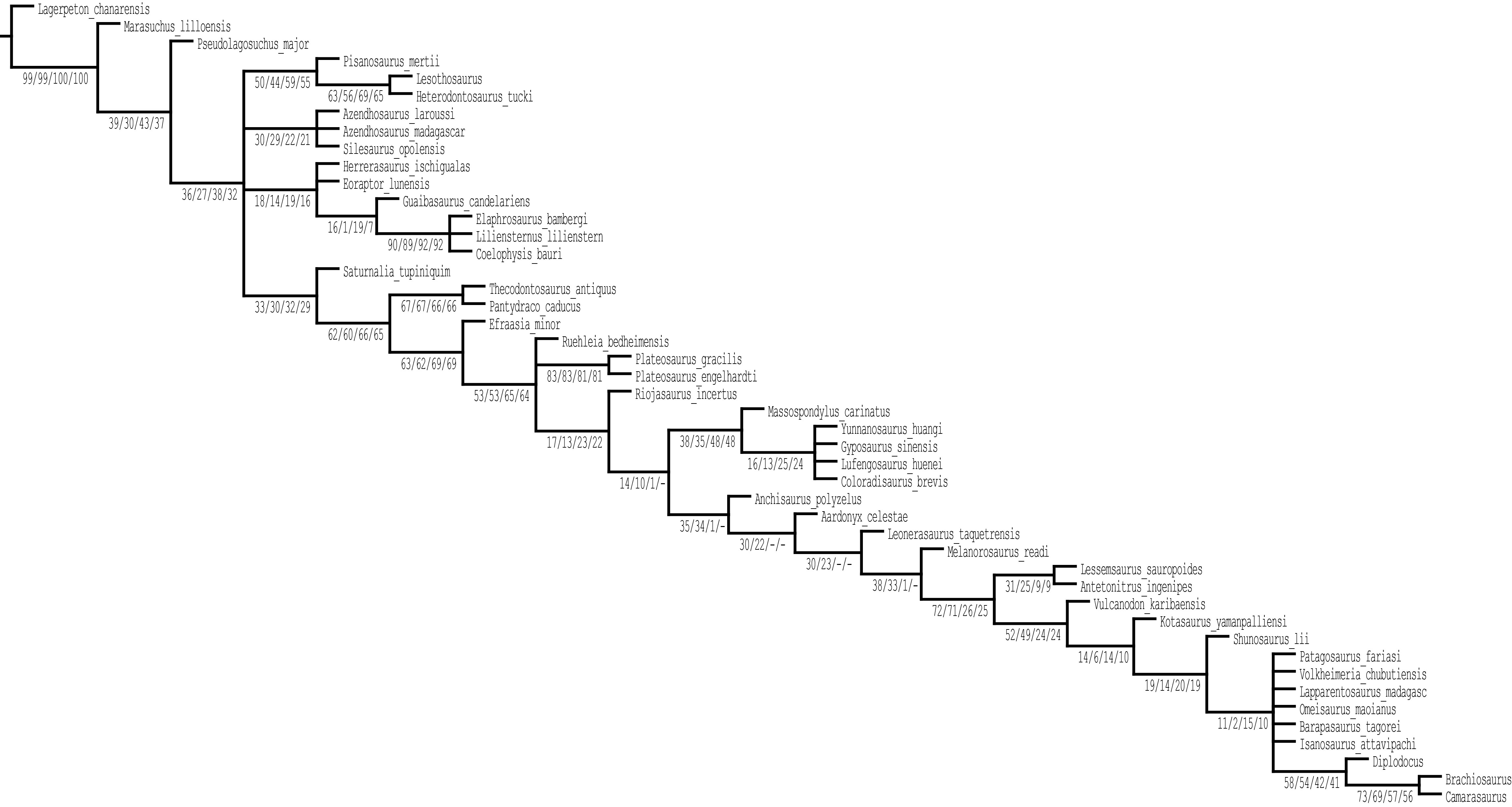
**

**2. Supplementary information to Figure 16**

The figure 16 represents the evolution of two relevant features in basal sauropodomorphs and basal sauropods. These are the presence of caudosacral and dorsosacral vertebrae (characters 150 and 151) and the optimization of mediolateral width of the femoral shaft (FML). This measure is taken as a proxy for body mass, given their tight linear correlation with body mass in a large number of terrestrial taxa. This approximation was used by Carrano (2005) to study the evolution of body size in Dinosauria (see Carrano 2005 for a more detailed explanation and justification of this approach). The FML was chosen not only due to its linear correlation with body mass but also because it represents the change in the eccentricity of the femoral shaft in the origin of Sauropoda. The FML was not included in the phylogenetic analysis but optimized in the most parsimonious trees.

**2.a Data.** The following table details the values of FML used for the sauropodomorphs in which this information was available. Most of these measures were taken from the extensive study on the evolution of dinosaur size conducted by Carrano (2005). Data on some taxa not included in that study were taken from the literature or directly measured in the specimens, as detailed in the table. If more than one specimen was available for a single taxon, the largest FML measurement was taken.

| **Taxa** | **FML (cm)** | **Source** |
| --- | --- | --- |
| *Marasuchus_lilloensis* | 0.51 | Carrano (2005) |
| *Pisanosaurus_mertii* | 0.7 | Carrano (2005) |
| *Eoraptor_lunensis* | 1.27 | Carrano (2005) |
| *Saturnalia_tupiniquim* | 1.7 | Langer (2003) |
| *Pantydraco_caducus* | 0.68 | Yates (2003) |
| *Thecodontosaurus_antiquus* | 2.67 | Benton et al. (2000) |
| *Efraasia_minor* | 6.51 | Due to the compression of the femur, FML was estimated as equal to FAP (Carrano 2006) of the specimen SMNS 17928 |
| *Ruehleia_bedheimensis* | 7.8 | MB.R.4753 |
| *Plateosaurus_engelhardti* | 9.08 | Carrano (2005) |
| *Plateosaurus_gracilis* | 6.69 | Carrano (2005) |
| *Coloradisaurus_brevis* | 6.18 | Carrano (2005) |
| *Massospondylus_carinatus* | 4.21 | Carrano (2005) |
| *Lufengosaurus_huenei* | 11.5 | Carrano (2005) |
| *Gyposaurus_sinensis* | 2.77 | Carrano (2005) |
| *Yunnanosaurus_huangi* | 6.0 | Carrano (2005) |
| *Riojasaurus_incertus* | 10.62 | Carrano (2005) |
| *Anchisaurus_polyzelus* | 4.2 | Carrano (2005) |
| *Aardonyx_celestae* | 9.1 | Yates et al. (2010) |
| *Leonerasaurus_taquetrensis* | 4.0 | MPEF-PV 1663 |
| *Melanorosaurus_readi* | 9.8 | NM QR3314 |
| *Antetonitrus_ingenipes* | 14.1 | BPI/1/4952 |
| *Lessemsaurus_sauropoides* | 12.4 | PVL 4822 |
| *Vulcanodon_karibaensis* | 17.4 | Carrano (2005) |
| *Isanosaurus_attavipachi* | 12.16 | Carrano (2005) |
| *Barapasaurus_tagorei* | 18.7 | Carrano (2005) |
| *Shunosaurus_lii* | 18.8 | Carrano (2005) |
| *Omeisaurus_maoianus* | 20.6 | Carrano (2005) |
| *Volkheimeria_chubutensis* | 7.5 | Carrano (2005) |
| *Patagosaurus_fariasi* | 25.5 | Carrano (2005) |
| *Diplodocus* | 18.6 | Carrano (2005) |
| *Camarasaurus* | 25.2 | Carrano (2005) |
| *Brachiosaurus* | 29.9 | Carrano (2005) |

**2.b Methods.** The FML continuous valued character was optimized using a modification of Wagner parsimony, as proposed by Goloboff et al. (2006) in TNT v. 1.0 (Goloboff et al., 2008a, b). Taxa for which no FML measurements were available were pruned from the MPTs prior to the optimization of the continuous valued character. The optimization of FML values shown at internal nodes in Figure 16 represents the most parsimonious ancestral reconstruction obtained in TNT. When the most parsimonious reconstruction yielded a range of possible values, the midpoint value of the ancestral reconstruction was taken to determine the size of the circle (and the grayscale value) shown in figure 16. The following tree shows the numerical value of the most parsimonious reconstructions obtained in TNT.

**3. Supplementary references**

Alifanov VR, Averianov AO (2003) *Ferganasaurus verzilini*, gen. et sp. nov., A new neosauropod (Dinosauria, Saurischia, Sauropoad) from the Middle Jurassic of Fergana Valley, Kirghizia. Journal of Vertebrate Paleontology 23: 358--372.

Bandyopadhyay, S, Gillette DD, Ray S, Sengupta DP (2010) Osteology of *Barapasaurus tagorei* (Dinosauria: Sauropoda) from the Early Jurassic of India. Palaeontology 53: 533-569.

Barrett PM, Upchurch P, Zhou X-D, Wang X-L (2007) The skull of *Yunnanosaurus huangi*  Young, 1942 (Dinosauria: Prosauropoda) from the Lower Lufeng Formation (Lower Jurassic) of Yunnan, China. Zoological Journal of the Linnean Society 150: 319—341.

Benton MJ, Juul L, Storrs GW, Galton PM (2000) Anatomy and systematics of the prosauropod dinosaur *Thecodontosaurus antiquus* from the Upper Triassic of southwest England. Journal of Vertebrate Paleontology 20: 71–102.

Bonaparte JF (1999) Evolución de las vértebras presacras en Sauropodomorpha. Ameghiniana 36: 115--187.

Buffetaut E, Suteethorn V, Cuny G, Tong H, Le Loeuff J, Khansubha S, Jongautcharlyakul S (2000) The earliest known sauropod dinosaur. Nature 407: 72--74.

Carrano MT (2005) Body-size evolution in the Dinosauria. In: Carrano MT, Gauden TJ, Blob RW, Wible JR, eds. Amnioti Paleobiology: Perspectives on the Evolution of Mammals, Birds and Reptiles. Chicago, IL: University of Chicago Press. pp 225–268.

Chatterjee S, Zheng Z (2002) Cranial anatomy of *Shunosaurus*, a basal sauropod dinosaur from the Middle Jurassic of China. Zoological Journal of the Linnean Society 136: 145--169.

Cooper MR (1984) A reassessment of *Vulcanodon karibaensis* Raath (Dinosauria: Saurischia) and the origin of the Sauropoda. Palaeontologia Africana 25: 203--231.

Dzik J (2003) A beaked herviborous archosaur with dinosaur affinities from the Early Late Triassic of Poland. Journal of Vertebrate Paleontology 23: 556--574.

Galton PM (1998) Saurischian dinosaurs from the Upper Triassic of England: *Camelotia* (Prosauropoda, Melanorosauridae) and *Avalonianus* (Theropoda, ?Carnosauria). Palaeontographica A 250: 155--172.

Galton PM, Upchurch P (2004) Prosauropoda. In: The Dinosauria 2^nd^ edition. Weishampel D, Dodson P, Osmolska H, eds. Berkeley, CA: University of California Press. pp. 232-258.

Gauffre F-X (1996) Phylogenie des dinosaures prosauropodes et etude d’un prosauropode du Trias Superieur d’Afrique Australe. Ph.D Thesis. Mus. Natl. d’ Hist. Nat. Paris. Vol. I, 156 pp.

Gauthier JA (1986) Saurischian monophyly and the origin of birds. In: Padian, K. (ed.). The Origin of Birds and the Evolution of Flight. Memoirs of the California Academy of Sciences 8: 1--55.

Goloboff PA, Farris JS, Kallersjo M, Oxelman B, Ramirez MJ, Szumik CA (2003) Improvements to resampling measures of group support. Cladistics 19: 324--332.

Goloboff PA, Mattoni CI, Quinteros AS (2006) Continuous characters analyzed as such. Cladistics 22: 1–13.

Goloboff PA, Farris JS, Nixon K (2008) TNT: Tree Analysis Using New Technology, vers. 1.1 (Willi Hennig Society Edition). Program and documentation, available at [www.zmuc.dk/public/phylogeny/tnt](http://www.zmuc.dk/public/phylogeny/tnt).

Goloboff PA, Farris JS, Nixon K (2008) TNT, a free program for phylogenetic analysis. Cladistics 24: 774-–786.

Jain SL, Kutty TS, Roy-Chowdhury T, Chatterjee S (1975) The sauropod dinosaur from the Lower Jurassic Koto Formation of India. Proceedings of the Royal Society of London B 188: 221--228.

Jain SL, Kutty TS, Roy-Chowdhury T, Chatterjee S (1979) Some characters of Barapasaurus tagorei, a sauropod dinosaur from the Lower Jurassic of Deccan, India. Proceedings of the IV International Gondwana Symposium 1: 204--216.

Langer MC (2003) The pelvic and hind limb anatomy of the stem-sauropodomorph *Saturnalia tupiniquim* (Late Triassic, Brazil) Paleobios 23: 1–40.

Langer MC (2004) Basal dinosaurs. In: The Dinosauria, 2^nd^ edition. D. Weishampel, H. Olsmolska (eds.). University of California Press.

Novas FE (1996). Dinosaur monophyly. Journal of Vertebrate Paleontology 16: 723—741

Pol D, Powell JE (2007) New information on *Lessemsaurus sauropoides* (Dinosauria: Sauropodomorpha) from the Upper Triassic of Argentina. Special Papers in Palaeontology 77: 223--243.

Raath MA (1972) Fossil vertebrate studies in Rhodesia: A new dinosaur (Reptilia: Saurischia) from near the Trias-Jurassic boundary. Arnoldia 5: 1--37.

Rauhut OWM (2003) The interrelationships and evolution of basal theropod dinosaurs. Special Papers in Palaeontology 69: 1--213.

Salgado L, Coria RA, Calvo JO (1997) Evolution of titanosaurid sauropods. I: Phylogenetic analysis based on the postcranial evidence. Ameghiniana 34: 3--32

Sereno PC (1999) The evolution of dinosaurs. Science 284: 2137-2147.

Sereno PC, Novas FE (1992) The complete skull and skeleton of an early dinosaur. Science 258: 1137--1140.

Sereno PC, Forster CA, Rogers RR, Monetta AM (1993) Primitive dinosaur skeleton from Argentina and the early evolution of Dinosauria. Nature 361: 64--66.

Tang F, Jin X-S, Kang X-M, Zhang G-J (2001) *Omeisaurus maoianus*, a complete Sauropoda from Jingyan, Sichuan. China Ocean Press, Beijing,128 pp. [In Chinese, English summary].

Upchurch P (1995) The evolutionary history of sauropod dinosaurs. Philosophical Transactions of the Royals Society of London B 349: 365--390.

Upchurch P (1998) The phylogenetic relationships of sauropod dinosaurs. Zoological Journal of the Linnean Society 124: 43--103.

Wedell MJ (2003) The evolution of vertebral pneumaticity in sauropods dinosaurs. Journal of Vertebrate Paleontology 23: 344--357.

Wilson JA (2002) Sauropod dinosaur phylogeny: critique and cladistic analysis. Zoological Journal of the Linnean Society 136: 217-276.

Wilson JA, Sereno PC (1998) Early evolution and higher-level phylogeny of sauropod dinosaurs. Memoirs of the Society of Vertebrate Paleontology 5: 1-68.

Yadagiri P (2001). The osteology of *Kotasaurus yamanpalliensis*, a sauropod dinosaur from the Early Jurassic Kota Formation of India. Journal of Vertebrate Paleontology 21: 242--252.

Yates AM (2003) A new species of the primitive dinosaur *Thecodontosaurus* (Saurischia: Sauropodomorpha) and its implications for the systematics of early dinosaurs. Journal of Systematic Palaeontology 1: 1–42.

Yates AM (2007) The first complete skull of the Triassic dinosaur *Melanorosaurus* Haughton (Sauropodomorpha: Anchisauria). Special Papers in Palaeontology 77: 9--55.

Yates AM (2010) A revision of the problematic sauropodomorph dinosaurs from Manchester, Connecticut and the status of *Anchisaurus* Marsh. Palaeontology 53: 739--752

Yates AM, Kitching JW (2003) The earliest known sauropod dinosaur and the first steps towards sauropod locomotion. Proceedings of the Royal Society of London B 270: 1753—1758.

Yates AM, Bonnan MF, Neveling J, Chinsamy A, Blackbeard MG (2010) A new transitional sauropodomorph dinosaur from the Early Jurassic of South Africa and the evolution of sauropod feeding and quadrupedalism. Proceedings of the Royal Society of London B 277: 787–794.

Zhang Y (1988) [The Middle Jurassic dinosaur fauna from Dashanpu, Zigong, Sichuan. Sauropod dinosaurs. Pt. 1. *Shunosaurus*.] Sichuan Sci. Tech. Publ. House, Chengdu. 89 pp. (In Chinese with English summary).

Zhang Y, Yang Z (1994) [A complete osteology of Prosauropoda in Lufeng Basin Yunnan China. *Jingshanosaurus*.] Yunnan Sci. Tech. Publ. House, Kunming. 100 pp. (In Chinese with English summary).
