# Supplementary material for: A New Sauropodomorph Dinosaur from the Early Jurassic of Patagonia and the Origin and Evolution of the Sauropod-type Sacrum
Source: PLoS One. 2011 Jan 26;6(1):e14572. doi: 10.1371/journal.pone.0014572 (PMC3027623; doi:10.1371/journal.pone.0014572)
Supplement: Table S1 — Selected measurements of Leonerasaurus taquetrensis (MPEF-PV 1663). (0.07 MB DOC) [file pone.0014572.s003.doc]

**Table S1.** Selected measurements of *Leonerasaurus taquetrensis* (MPEF-PV 1663).

| **Element** | **Dimension** | **Length [mm]** |
| --- | --- | --- |
| Axis | Centrum length* | 32 |
|  | Centrum width | 22.5 |
|  | Centrum height | 21.1 |
| Cervical 3 | Centrum length | 40.8 |
|  | Centrum width | 16.1 |
|  | Centrum height | 18.4 |
|  | Neural arch height | 15.9 |
| Cervical 4 | Centrum length | 47.1 |
|  | Centrum width | 17.6 |
|  | Centrum height | 18.7 |
|  | Neural arch height | 24.3 |
| Cervical 5 | Centrum length | 50.8 |
|  | Centrum width | 21.5 |
|  | Centrum height | 18.5 |
|  | Neural arch height | 23.8 |
| Dorsal 1 | Centrum length | 40.1 |
|  | Centrum width | 24.6 |
|  | Centrum height | 24.6 |
|  | Neural arch height | 23.6 |
| Dorsal 2 | Centrum length | 38 |
|  | Centrum width | 25.2 |
|  | Centrum height | 26.4 |
|  | Neural arch height | 26.3 |
| Dorsal 3 | Centrum length | 41.8 |
|  | Centrum width | 22.2 |
|  | Centrum height | 27.6 |
| Dorsal 4 | Centrum length | 42.3 |
|  | Centrum width | 24.6 |
|  | Centrum height | 28.5 |
| Mid dorsal | Centrum length | 42.7 |
|  | Centrum width | 34 |
|  | Centrum height | 37.7 |
|  | Neural arch height | 31.7 |
| Dorsosacral | Centrum length | 46.9 |
|  | Centrum width | 32.6 |
|  | Centrum height | 28.6 |
| Primordial sacral 1 | Centrum length | 43 |
|  | Centrum width | 30.1 |
|  | Centrum height | 26.7 |
| Primordial sacral 2 | Centrum length | 43 |
|  | Centrum width | 28.3 |
|  | Centrum height | 25.6 |
| Caudosacral | Centrum width | 27.1 |
|  | Centrum height | 30 |
| Scapula | Proximodistal length* | 166.1 |
|  | Minimum shaft width | 26.1 |
|  | Dorsal blade width* | 36.1 |
| Humerus | Proximodistal length | 202.8 |
|  | Proximal width | 56.7 |
|  | Minimum shaft width | 22.15 |
|  | Distal width | 61.9 |
| Ilium | Anteroposterior length* | 132.2 |
| Ischium | Length* | 146.1 |
| Metatarsal I | Proximodistal length | 67 |
|  | Proximal height | 24.6 |
|  | Proximal width | 12.8 |

Measurements of complete vertebrae are included here. The centrum height and width are taken at the posterior articular surfaces (except for the caudosacral that was measured at the anterior end, because it lacks its posterior end). The neural arch height does not include the neural spine and is taken from the posterior end of the neurocentral suture to the base of the neural spine. The mid dorsal measurements correspond to the posteriormost element of the three articulated vertebrae. Measurements are taken as preserved, with an asterisk (*) indicating those elements that are incomplete.
